# Dynamic range expansion leads to establishment of a new, genetically distinct wolf population in Central Europe

**DOI:** 10.1038/s41598-019-55273-w

**Published:** 2019-12-12

**Authors:** Maciej Szewczyk, Sabina Nowak, Natalia Niedźwiecka, Pavel Hulva, Renata Špinkytė-Bačkaitienė, Klára Demjanovičová, Barbora Černá Bolfíková, Vladimír Antal, Viktar Fenchuk, Michał Figura, Patrycja Tomczak, Przemysław Stachyra, Kinga M. Stępniak, Tomasz Zwijacz-Kozica, Robert W. Mysłajek

**Affiliations:** 1 Institute of Genetics and Biotechnology, Faculty of Biology, University of Warsaw, Pawińskiego 5a, 02-106 Warsaw, Poland; 2 Association for Nature “Wolf”, Twardorzeczka, Cynkowa 4, 34-324 Lipowa, Poland; 3 Department of Vertebrate Ecology and Zoology, Faculty of Biology, University of Gdańsk, Wita Stwosza 59, 80-308 Gdańsk, Poland; 4 Faculty of Science, Charles University in Prague, Viničná 7, 128 43 Prague, Czech Republic; 5 Faculty of Science, University of Ostrava, Chittussiho 10, 170 00 Ostrava, Czech Republic; 6 Vytautas Magnus University, K. Donelaičio 58, 44248 Kaunas, Lithuania; 7 Department of Animal Science and Food Processing, Faculty of Tropical AgriSciences, Czech University of Life Sciences Prague, Kamýcká 129, Prague 6, 165 00 Czech Republic; 8 State Nature Conservancy of Slovak Republic, Tajovského 28B, 974 01 Banská Bystrica, Slovakia; 9 APB-BirdLife Belarus, Engelsa 34A - 1, 220030 Minsk, Belarus; 10 Institute of Romance Studies, Faculty of Modern Languages and Literature, Adam Mickiewicz University in Poznań, Al. Niepodległości 4, 61-874 Poznań, Poland; 11 Roztocze National Park, Plażowa 2, 22-470 Zwierzyniec, Poland; 12 Tatra National Park, Kuźnice 1, 34-500 Zakopane, Poland

**Keywords:** Conservation biology, Structural variation

## Abstract

Local extinction and recolonization events can shape genetic structure of subdivided animal populations. The gray wolf (*Canis lupus*) was extirpated from most of Europe, but recently recolonized big part of its historical range. An exceptionally dynamic expansion of wolf population is observed in the western part of the Great European Plain. Nonetheless, genetic consequences of this process have not yet been fully understood. We aimed to assess genetic diversity of this recently established wolf population in Western Poland (WPL), determine its origin and provide novel data regarding the population genetic structure of the grey wolf in Central Europe. We utilized both spatially explicit and non-explicit Bayesian clustering approaches, as well as a model-independent, multivariate method DAPC, to infer genetic structure in large dataset (881 identified individuals) of wolf microsatellite genotypes. To put the patterns observed in studied population into a broader biogeographic context we also analyzed a mtDNA control region fragment widely used in previous studies. In comparison to a source population, we found slightly reduced allelic richness and heterozygosity in the newly recolonized areas west of the Vistula river. We discovered relatively strong west-east structuring in lowland wolves, probably reflecting founder-flush and allele surfing during range expansion, resulting in clear distinction of WPL, eastern lowland and Carpathian genetic groups. Interestingly, wolves from recently recolonized mountainous areas (Sudetes Mts, SW Poland) clustered together with lowland, but not Carpathian wolf populations. We also identified an area in Central Poland that seems to be a melting pot of western, lowland eastern and Carpathian wolves. We conclude that the process of dynamic recolonization of Central European lowlands lead to the formation of a new, genetically distinct wolf population. Together with the settlement and establishment of packs in mountains by lowland wolves and vice versa, it suggests that demographic dynamics and possibly anthropogenic barriers rather than ecological factors (e.g. natal habitat-biased dispersal patterns) shape the current wolf genetic structure in Central Europe.

## Introduction

Range expansions in a heterogeneous environment can diversely shape population genetic diversity and structure of a species^1–3^. Founder effects during expansions are one of the main drivers of genetic structuring, leading to establishment of new, genetically distinct populations^4–8^. On the other hand, rapid population expansion may promote gene flow and admixture^9,10^, sometimes resulting in a nearly homogenous genetic structure^11^.

Wolves were historically the most widespread large terrestrial carnivores in the northern hemisphere. However, centuries of persecution led to the collapse of many wolf populations, resulting in global reduction in its historical range to 68%, including most of western and central Europe^12^. In Poland, wolves were never completely extirpated despite an intentional extermination program conducted for several decades after the Second World War. However, the population was severely reduced to less than a hundred individuals that survived at the eastern edges of the country^13^.

After implementation of strict protection across the whole Poland in 1998 wolves started to repopulate the vast forest tracts west of the Vistula river. The first phase of recolonization was characterized by jump dispersal leading to establishment of a few packs in distant locations, and a relatively slow pace of population recovery. However, in the second phase the dispersal pattern shifted to a mixture of diffusion and jump dispersal, resulting in the creation of packs in close vicinity to existing groups and much more dynamic population growth^14^. The population inhabiting Western Poland (WPL), together with wolves recolonizing Germany, western Czech Republic, Denmark and the Netherlands, is considered to be a distinct management unit called the Central European wolf population^15^. However, results of genetic studies of Polish wolf populations^16^ that were performed during the first stage of WPL recolonization and included samples collected from 2002 to 2009 in all nine wolf packs living at the time west of the Vistula river, suggested that wolves inhabiting Western Poland and Eastern Germany constitute one population with those from the north-eastern part of Poland, which are classified as belonging to the Baltic population (*sensu* Chapron *et al*.^15^).

Currently, wolves have already colonized most suitable habitats in WPL^17^. Earlier study^16^ indicated concordant mitochondrial and nuclear DNA structuring between Polish lowland and Carpathian wolves that might be attributed either to landscape fragmentation and dispersal barriers or to ecological differences between studied populations resulting in natal habitat-biased dispersal. However, there are documented cases of long distance wolf dispersals in human-dominated European landscapes, with large number of dispersal barriers^18–20^. Moreover, several cases of the settlement of lowland wolves in mountainous regions were reported^20^, putting both hypotheses into question. Furthermore, there are some examples of cryptic structuring of wolf populations that cannot be simply explained by geographic and anthropogenic barriers to dispersal or by ecological factors^21–24^, thus highlighting that mechanisms shaping wolf genetic structure are still not fully understood. The currently recolonized areas in WPL are stratified in terms of anthropogenic pressure and landscape characteristics, as they are composed mostly of highly forested lowlands, but also some uplands, the Sudetes mountain range and areas dominated by arable lands^17,25^. Hence, the recolonization process is a unique opportunity to study the relationships between habitat selection and genetic origins of wolves.

**Figure 1:**
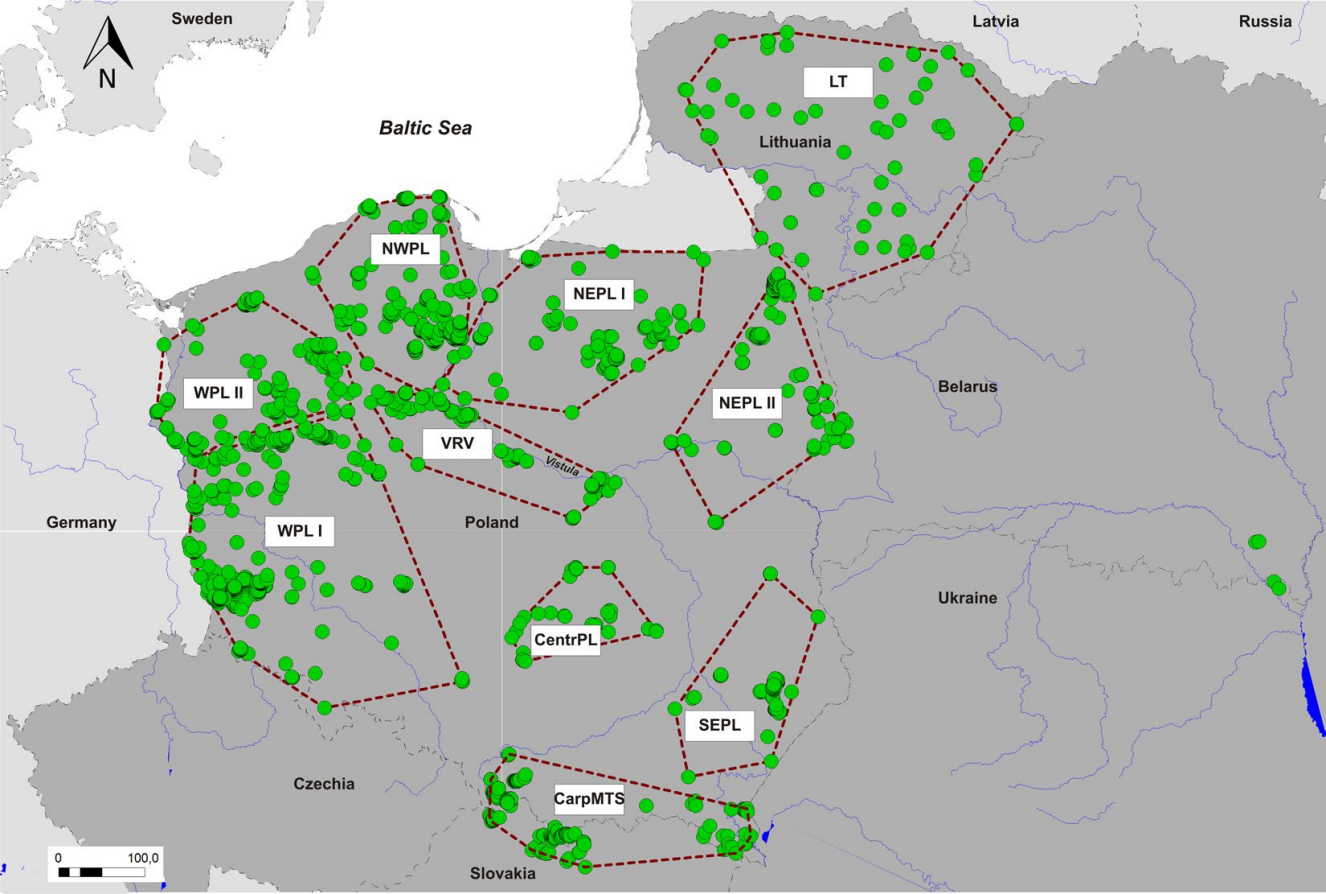
Distribution of genetic samples used in present study (green dots). State borders are denoted with gray dashed lines, main rivers with blue solid lines and borders of the predefined regions with red dashed lines. Countries belonging to study area shaded in dark gray. WPL_I - Western Poland, south-central part (including Sudetes Mountains); WPL_II - Western Poland, north-central part; NWPL - North-Western Poland; VRV - Vistula River Valley; CentrPL - Central Poland; NEPL_I - North-Eastern Poland, north-central part; NEPL_II - North-Eastern Poland, easternmost part; SEPL - South-Eastern Poland; LT – Lithuania; CarpMTS - Carpathian Mountains.

Taking advantage of a large number of non-invasive samples collected over eight years in WPL, central, north-eastern, eastern Poland, Lithuania, Belarus and the Carpathians (Fig. 1), we examined the spatial genetic population structure of Central European wolves, with special focus on the understudied, recently established population west of the Vistula river. We combined different Bayesian clustering and multivariate methods to identify genetic groups and examined patterns of partition of genetic diversity among those groups and geographic regions. We assumed that despite its presumable eastern origin, the wolf population in Western Poland could emerge as a separate group, because the population was initiated in result of the long-distance (jump) dispersal of few individuals which formed first packs in WPL^14^ and such demographic events could result in allele frequencies strikingly different from source populations due to founder effect and allele surfing^26,27^. We also attempted to update delimitation of management units for wolves in Central Europe, taking under consideration results of all recent analysis of the wolf genetic structure from this region.

## Results

### Genetic variability

Multilocus consensus genotypes of sufficient quality (at least 9 genotyped loci) were obtained for 1514 samples, in which we identified in total 881 wolf individuals (on average 1.72 samples/individual, range 1–18). Identified putative wolf-dog hybrids were not included in this dataset (see Supplementary Methods and Suppl. Fig. S1 for details). The overall mean allelic drop-out rate was 0.036 and ranged from 0.014 in locus FH2088 to 0.105 in FH2017. Null alleles were not detected. The cumulative probability of identity (P _ID_) for all genotypes was very low and equaled 4.9 × 10^–12^ (range 1.2 × 10^−9^–1.3 × 10^−13^ depending on a region) for unrelated individuals and 2.4 × 10^−5^ (range 1.3 × 10^−4^–9.0 × 10^−6^) for full siblings.

When we estimated basic microsatellite variability statistics among sampling regions (summarized in Table 1; details per each locus can be found in Supplementary Table S1) all loci were polymorphic in each region. The mean number of alleles, allelic richness and observed heterozygosity were the highest in Lithuania (LT; 6.846, 6.110, and 0.738, respectively), while regions with the lowest H_O_ were NEPL I and WPL I (0.589 and 0.597, respectively). Generally, allelic richness was significantly lower in the recently recolonized regions (mean 4.333 in grouped WPL I, WPL II, NWPL, VRV and CentrPL regions compared to 5.171 in grouped NEPL I, NEPL II, LT and SEPL; p = 0.015), but mean observed heterozygosity did not differ significantly compared to eastern lowland regions (p = 0.08). Inbreeding coefficient (F_IS_) values were low and ranged from −0.052 to 0.054.

**Table 1:** Microsatellite summary statistics for the 10 predefined geographic regions.

Region	N	Na	A_R_	H_O_	H_E_	uH_E_	F_IS_	PA
WPL I	255	5.615	4.331	0.597	0.592	0.593	−0.007	0
WPL II	103	5.385	4.583	0.645	0.645	0.649	0.007	0
NWPL	104	6.154	4.954	0.627	0.644	0.647	0.031	2
VRV	51	4.615	4.062	0.614	0.577	0.583	−0.052	0
CentrPL	22	5.385	5.356	0.693	0.671	0.687	−0.008	1
NEPL I	75	6.000	5.051	0.589	0.618	0.622	0.054	1
NEPL II	60	6.231	5.514	0.694	0.696	0.702	0.011	1
LT	58	6.846	6.112	0.738	0.733	0.740	0.002	1
SEPL	34	5.385	4.981	0.655	0.656	0.667	0.017	0
CarpMTS	112	6.923	5.698	0.650	0.675	0.678	0.042	3
total/mean	874	5.854	5.658	0.650	0.651	0.657	0.010	—

N – number of individuals, Na – mean number of alleles, A_R_ – rarefied allelic richness, H_O_ – observed heterozygosity, H_E_ – expected heterozygosity, uH_E_ – unbiased expected heterozygosity, F_IS_ - inbreeding coefficient, PA - number of private alleles.

Pairwise F_ST_ values between the 10 predefined geographic regions (Fig. 1) ranged from 0.014 to 0.172 (Table 2). All pairwise values remained significant after Bonferroni correction (based on 1000 permutations; p < 0.05), but for most regions in western and northern Poland we observed F_ST_ values generally considered as low (<0.05^28^). The highest recorded were the pairwise F_ST_ values between the Carpathians and the recently recolonized regions in western and northern Poland. Also the SEPL region was relatively highly differentiated from other lowland regions, whereas a relatively low level of differentiation was found between Lithuania and CarpMTS and SEPL regions (0.085 and 0.055, respectively). Pairwise R_ST_ values were generally higher than corresponding F_ST_ values, as could be expected in case of highly polymorphic microsatellite markers^28^. However, the observed general pattern was very similar, with very low (often insignificant) differentiation between western regions, moderate to high differentiation between western and eastern lowlands and very high pairwise R_ST_ values between CarpMTS and western regions, especially WPL I.

**Table 2:** Pairwise F_ST_ (below diagonal) and R_ST_ (above diagonal) values between 10 predefined geographic regions.

Region	WPL I	WPL II	NWPL	VRV	CentrPL	NEPL I	NEPL II	LT	SEPL	CarpMTS
WPL I	*	**0.022**	**0.053**	**0.029**	**0.041**	**0.046**	**0.088**	**0.224**	**0.188**	**0.283**
WPL II	**0.023**	*	*0.006*	*0.000*	*0.010*	*0.008*	**0.021**	**0.103**	**0.082**	**0.168**
NWPL	**0.030**	**0.014**	*	**0.036**	*0.018*	*0.009*	**0.016**	**0.092**	**0.084**	**0.164**
VRV	**0.036**	**0.030**	**0.045**	*	**0.083**	**0.039**	**0.059**	**0.223**	**0.255**	**0.214**
CentrPL	**0.036**	**0.019**	**0.022**	**0.042**	*	*0.023*	*0.018*	**0.105**	**0.078**	**0.117**
NEPL I	**0.035**	**0.025**	**0.021**	**0.039**	**0.036**	*	*0.009*	**0.105**	**0.094**	**0.163**
NEPL II	**0.057**	**0.033**	**0.035**	**0.056**	**0.029**	**0.032**	*	**0.040**	**0.042**	**0.111**
LT	**0.087**	**0.051**	**0.049**	**0.082**	**0.034**	**0.053**	**0.017**	*	**0.073**	**0.082**
SEPL	**0.110**	**0.080**	**0.078**	**0.119**	**0.052**	**0.094**	**0.070**	**0.055**	*	**0.132**
CarpMTS	**0.172**	**0.139**	**0.135**	**0.167**	**0.085**	**0.146**	**0.123**	**0.085**	**0.130**	*

Statistically significant values are bolded.

**Figure 2:**
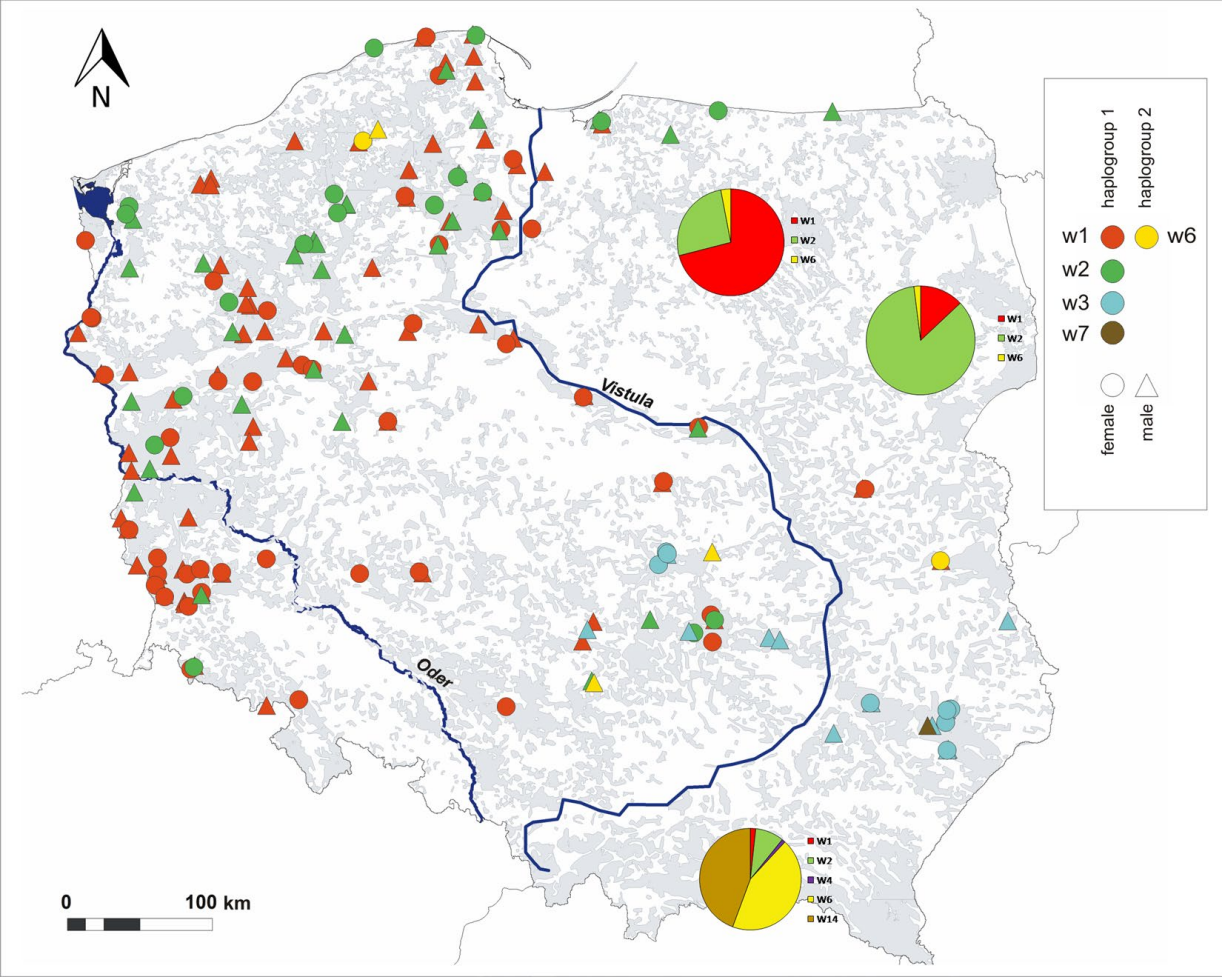
Distribution of mtDNA control region haplotypes of Polish wolves sequenced in this study. Additionally, for regions where the wolf range has not significantly changed recently, previously published haplotype frequencies are presented as circle diagrams (north-eastern Poland - Czarnomska *et al*., 2013; Carpathian Mountains - Hulva *et al*., 2018). Forest (shaded in gray) and main rivers are denoted.

We found relatively low mtDNA diversity in most of the recently recolonized areas (WPL I and II, NWPL and VRV), with almost all analyzed individuals bearing haplotypes w1 and w2, both belonging to haplogroup 1^29^ (Fig. 2). The only exceptions were two closely related wolves discovered in the NWPL region that had the w6 haplotype, which is common in the Carpathians. South-western regions together with Sudetes Mts. were strongly dominated by the w1 haplotype, while in north-western Poland frequencies of the w1 and w2 haplotype were similar (55% and 42%, respectively). Predominantly we observed an island pattern of mtDNA haplotypes distribution (e. g. the island of the w2 haplotype in the WPL II region or several islands of the w1 haplotypes in WPL I and VRV), but in NWPL, the haplotypes were more evenly distributed.

We recorded the highest mtDNA diversity in the relatively small CentrPL region that lacks big, continuous forest tracts. We detected there two packs bearing Carpathian w6 haplotype. It was also the only region west of the Vistula river where the w3 haplotype was present. In three packs from this region in which we identified breeding pairs, breeding females had different mtDNA haplotypes than males.

In the SEPL region the rare elsewhere w3 haplotype was dominant. The only exception were two individuals from the northernmost part of the region (not connected directly with the main SEPL forest tracts) carrying the w1 and w6 haplotypes and one individual with the w7 haplotype typical for eastern Belarus^21^. It was the only case of the w7 in our dataset. We have not detected there any individual with w2 haplotype which was reported to be the most frequent haplotype in central and northern parts of Eastern Poland^16^.

### Genetic clustering analyses

Bayesian clustering analysis with STRUCTURE software revealed that in our whole dataset of 881 wolf individuals with an origin from the Central European, Baltic and Carpathian populations (*sensu* Chapron *et al*.^15^) the optimum number of genetic clusters is three (as determined by the Evanno method^30^; Suppl. Fig. S2A,B). At K = 2 we did not observe clear separation of Carpathian and lowland wolves as reported previously^16,20^, as individuals from eastern Poland and Lithuania clustered mostly with the Carpathians. However, at K = 3 those individuals formed a distinct cluster, resulting in clear partitioning to Carpathian, eastern lowland and western populations. These results were supported by DAPC analysis, where DA1 clearly separated Carpathian wolves from all other regions, while DA2 differentiated eastern lowland wolves (regions SEPL and LT) from those from the western lowlands (Suppl. Fig. S2C).

STRUCTURE results obtained for higher than K = 3 values suggested the presence of an additional substructure inside the western lowland cluster (e.g. a distinct cluster corresponding to one big forest tract in WPL I region at K = 5). However, such a pattern was not observed in the DAPC analysis. As STRUCTURE was designed to place individuals into Hardy–Weinberg/linkage equilibrium populations^31^ and DAPC is model-independent^32^, we assumed that this discrepancy between the results of both methods is caused by the presence of a large number of closely related individuals in our dataset that could interfere with Bayesian clustering algorithms^33^. Thus, we reduced this possible bias by balancing number of analyzed individuals per pack. Based on field data and genetic relatedness analysis, we identified 80 wolf family groups with ≥3 sampled individuals. When possible, we inferred the most likely breeding pairs and these parents were retained in the dataset, while their offspring were removed, as was performed in other studies^34^. Otherwise, we retained one random male and one random female with the best quality genotypes per pack. This procedure led to reduction of the dataset from 881 to 451 individuals. The subset from the region WPL I, where the largest groups of kin were present due to intensive long-term samples collecting was reduced over 3-fold (from 255 to 81 individuals). By contrast, the smallest subsets from regions CentrPL and SEPL were the least affected (27% and 34% reduction, respectively). We also recorded that some adult wolves killed in traffic accidents in Western Poland were not related to local packs or died outside of the known wolf range. Thus, we assumed that they were floaters and hence cannot be assigned to local populations. A detailed scrutiny of genetic and field data led to the identification of 27 such individuals (20 males and 7 females). They were retained in non-spatial analyses (as a separate group “putative dispersers”), but excluded from spatial analyses. Furthermore, to improve sampling coverage of eastern populations, we added to analysis samples from western Belarus and from both Belarusian and Ukrainian parts of the Chernobyl Exclusion Zone (herein region BY-UA).

**Figure 3:**
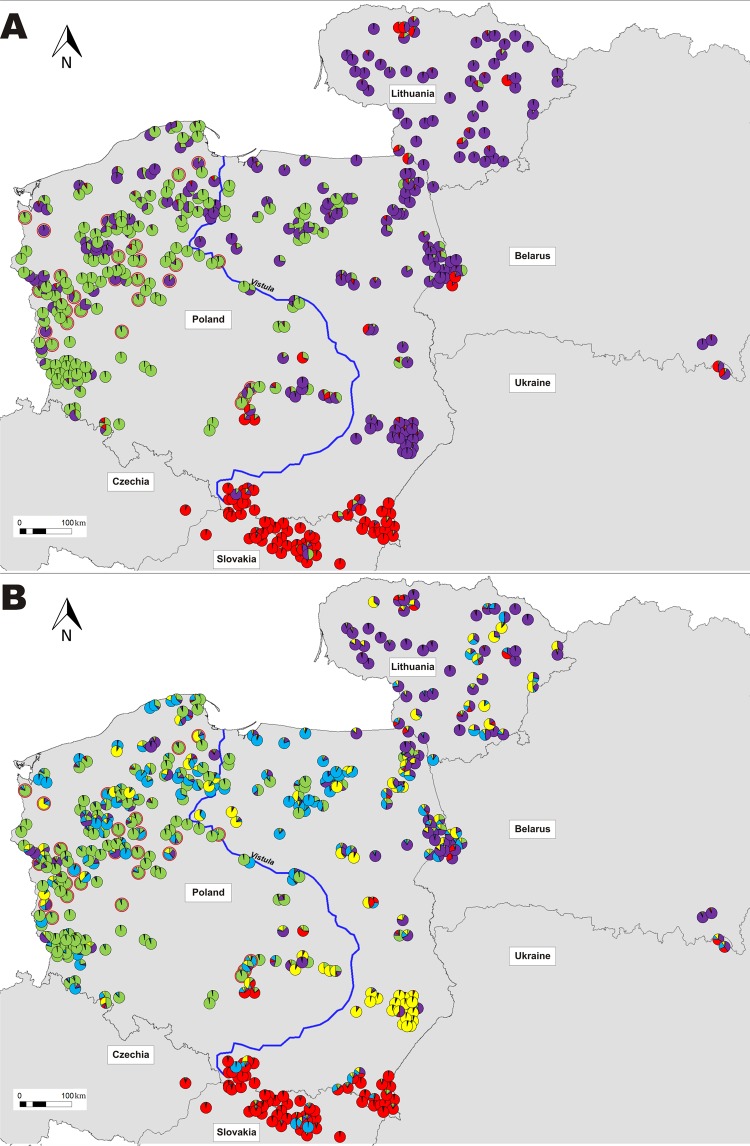
Individual cluster membership proportions of wolves sampled in this study, according to the STRUCTURE analysis for K = 3 (panel A) and K = 5 (panel B). Putative dispersers (adult road-killed wolves that were not assigned to local packs) are marked with red edging.

Results of STRUCTURE analysis of this reduced dataset were generally congruent with those obtained for the whole dataset, as K = 3 again obtained the highest support from the Evanno method (Suppl. Fig. S3A,B). The three identified clusters (Fig. 3A) corresponded to the Carpathian Mountains, eastern lowlands (regions LT, BY-UA, NEPL II and SEPL) and western lowlands (regions WPL I and VRV, most of WPL II, NWPL and NEPL I). The CentrPL region was the most diverse, with similar proportions of each cluster and several admixed individuals.

However, a determination of “true” number of clusters from STRUCTURE runs is not always straightforward. Relying solely on the ∆K method may lead to underestimation of population genetic structure^35^. The observed log-likelihood values still increased substantially for K ≥ 4, indicating the existence of additional substructure. Thus, we performed a semi-hierarchical analysis. First, we re-performed the analysis after exclusion of all individuals assigned to the Carpathian cluster, as 1) our main aim was to explore the genetic structure in the recently recolonized lowlands, and 2) substructuring of the Carpathian population was studied before^20^. Then the highest change in ΔK was observed at K = 2, reflecting the partitioning of lowland wolves to eastern and western populations (individual assignments were congruent with those at K = 3 in previous analysis). However, a substantial ΔK change was observed also at K = 4 (Suppl. Fig. S3C,D). Likewise it was the highest K that gave the same clustering pattern in all 10 iterations. Further hierarchical analysis, in which each of the clusters identified at K = 2 was analyzed separately, confirmed this result: cluster 1 (“western”) did not have any meaningful subpartition, indicating K = 1, while for cluster 2 (“eastern”) the best number of K as determined by the Evanno method was 3 (Suppl. Fig. S4). Patterns of individual assignments were very similar as for K = 4 in the analysis excluding Carpathians and for K = 5 in the general analysis. At this level of population partitioning (Fig. 3B), Carpathian wolves again formed a nearly homogeneous cluster and most of areas west of the Vistula river were dominated by wolves assigned to cluster 1 (western). However, eastern lowland wolves were split into three clusters: south-eastern (cluster 5 - dominant in the SEPL region, only small islands in other regions), north-eastern (cluster 2 - dominant in regions LT, NEPL II and BY-UA) and northern (cluster 4 -dominant in the NEPL I region; relatively high frequency also in regions NWPL and WPL II).

**Figure 4:**
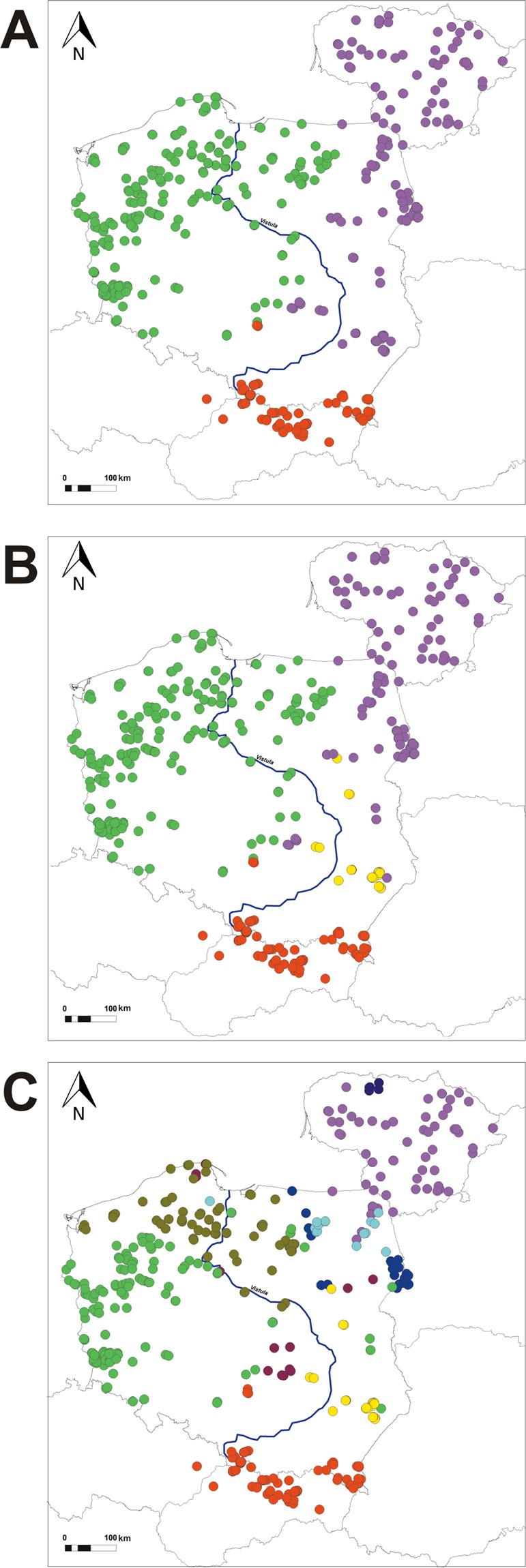
Spatial projection of GENELAND results. Panel A - uncorrelated allele frequency model, K = 3; panel B - uncorrelated allele frequency model, K = 4; panel C - correlated allele frequency model, K = 9.

Next, we used a spatially-explicit clustering method implemented in GENELAND to infer the spatial structure in the dataset of 434 individuals (the same as in STRUCTURE analyses except that the putative dispersers were not included). In the first GENELAND runs we observed an appearance of “ghost populations”. Thus, we afterwards excluded 4 individuals from the Chernobyl zone – a region >300 km distant to our main study area. In subsequent analysis utilizing an uncorrelated allele frequency model, in 6 out of 10 runs GENELAND identified 3 clusters, while in the remaining runs – 4 clusters. On the other hand, two runs with the highest posterior probability were those identifying K = 4. Notably, comparison of individual assignments revealed that results of all of the runs were generally congruent, with the only exception being that south-eastern wolves that formed a distinct cluster at K = 4, at lower K were clustered together with individuals from the north-east (Fig. 4A,B, Suppl. Fig. S5). Assignments to the western and Carpathian clusters were the same in all runs. Next, we performed analysis using the correlated model. We observed a very high variance between runs, with identified K varying from 7 to 13 and differences in individual assignments even between runs with the same selected K value. In iterations with K ≥ 10, the software identified several meaningless populations corresponding to few, usually closely related individuals. However, some patterns were reproducible in all runs, as the observed split of western wolves to south-western and north-western clusters. Results of the run identifying K = 9, which had the highest mean posterior probability density, are shown on Fig. 4C.

**Figure 5:**
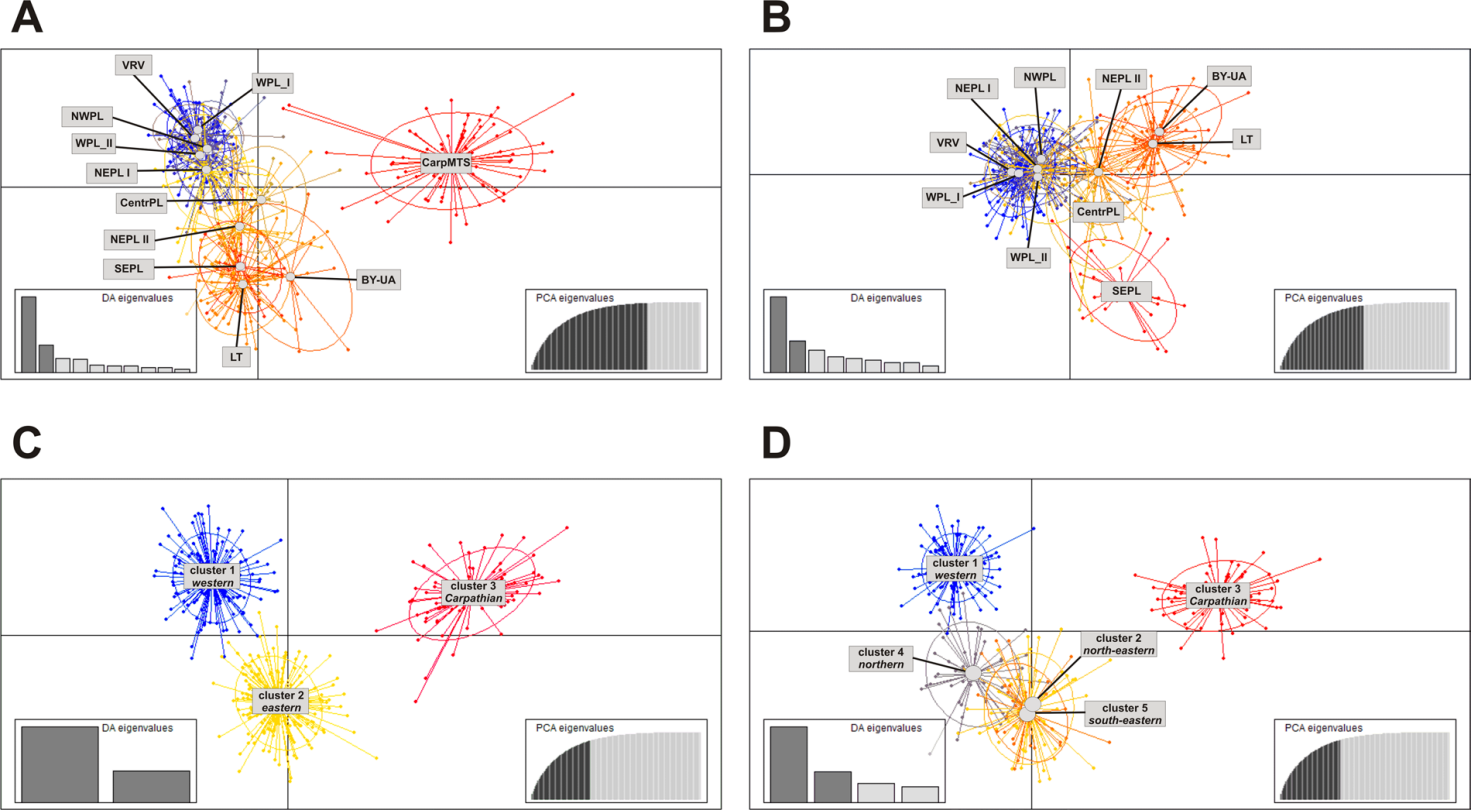
Scatterplots representing results of DAPC analyses. Panels A,B – analysis of predefined geographic regions (**A** – all regions analyzed, **B** – analysis excluding Carpathians), panels C,D – analysis of clusters identified in STRUCTURE (**C** – at K = 3, **D** – at K = 5).

Finally, we performed DAPC to infer genetic distance between the predefined geographic regions and clusters identified by STRUCTURE. In the analysis including all regions (but with putative dispersers excluded), Carpathian wolves were clearly separated from all lowland wolves by DA1, while DA2 reflected the west-east division in lowlands (Fig. 5A). Regions LT, BY-UA and SEPL formed one group on DA2 axis, while WPL I, WPL II, NWPL, VRV and NEPL I another. NEPL II and CentrPL were plotted in between. Analysis not including Carpathians gave similar results in terms of east-west differentiation, but additionally clearly separated the SEPL region from other eastern regions (Fig. 5B). Next, we used DAPC to verify the STRUCTURE results. In this analysis, we took into account only wolves assigned to a given cluster with q > 0.7 while individuals identified as admixed (N = 54 in case of K = 3 and N = 119 for K = 5) were excluded. The three main clusters identified in STRUCTURE were clearly separated by DAPC (Fig. 5C). However in case of K = 5, the south-eastern cluster largely overlapped with the north-eastern and the northern cluster was identified as an intermediary between western and north-eastern clusters (Fig. 5D). The latter result was concordant with identified between-cluster F_ST_ values, where the northern cluster showed a relatively low distance to both western and north-eastern clusters (Table 3). However, the south-eastern cluster was found to be at least moderately differentiated from all other groups. That was confirmed by additional DAPC analysis, where DA3 separated cluster 5 from the other clusters (Suppl. Fig. S6).

**Table 3:** Pairwise F_ST_ (below diagonal) and R_ST_ (above diagonal) values between clusters identified in STRUCUTRE analyses.

clusters identified at K = 3					
	western		eastern		Carpathian
western	*		**0.134**		**0.394**
eastern	**0.064**		*		**0.219**
Carpathian	**0.174**		**0.099**		*
**clusters identified at K = 5**					
	**western**	**northern**	**north-eastern**	**south-eastern**	**Carpathian**
western	*	**0.081**	**0.205**	**0.209**	**0.416**
northern	**0.093**	*	**0.134**	**0.197**	**0.347**
north-eastern	**0.097**	**0.081**	*	**0.078**	**0.156**
south-eastern	**0.107**	**0.121**	**0.070**	*	**0.308**
Carpathian	**0.189**	**0.167**	**0.100**	**0.145**	*

Statistically significant values are bolded.

Having identified the main wolf genetic groups and their geographic distribution, we investigated the rate of contemporary gene flow between them using BayesAss 3.0^36^. Firstly, we analyzed the rate and direction of gene flow between the areas recolonized by wolves during the last two decades (regions WPL I, WPL II, NWPL, VRV and CentrPL treated as one population) and those permanently occupied by wolves in the eastern lowlands (NEPL I, NEPL II, LT, BY-UA and SEPL) and in the Carpathians, roughly reflecting STRUCTURE clustering at K = 3. Concordantly with the STRUCTURE results, we found a moderate level of bidirectional gene flow between eastern populations and recolonized regions, low gene flow from lowlands to the Carpathians and from the Carpathians to eastern lowlands, and almost no gene flow from the Carpathians to western lowlands (Table 4). We performed also an analysis where, as suggested by GENELAND (at K = 4) and STRUCTURE (at K = 5) clustering, the SEPL region was treated as a discrete population. It suggested moderate immigration from both western and north-eastern population into the south-east, but much lower gene flow in the opposite direction (Suppl. Table S2). However, these results must be treated with caution due to low sample size of the south-eastern genetic group, as inequalities in sample sizes are known to affect BayesAss results^37^. As the STRUCTURE and DAPC results suggested the CentrPL region may be a „melting pot” of different wolf populations and the NEPL I region is a transition zone between the eastern and western genetic group, we reanalyzed the data after exclusion of the two aforementioned regions, aiming to assess the rate of long distance gene flow. As expected, the estimated migration rates were lower than in the analysis including transition zones (Table 4, Suppl. Table S2). Interestingly, while the estimate of gene flow from the east to the west was only about 30% lower than in variant with transition zones included, in the opposite way the estimate was over 3-fold lower, suggesting that wolves from Western Poland do disperse eastwards, but rarely further than to the NEPL I region. To investigate it further, we performed additional analyses where the putative transition zones (i.e. NEPL I and CentrPL regions) were defined as separate groups (Suppl. Table S3) or for all 11 predefined geographic regions treated as discrete populations (Suppl. Table S4). Although the exact values of estimated migration rates must be treated with caution due to unequal sample sizes, resulting in wide 95% confidence intervals, the general trend was congruent in all analyses: high rate of eastward gene flow from western regions to the “transition zones”, moderate westward gene flow from the NEPL I region and moderate dispersal from the easternmost regions to both the “transition zones” and Western Poland.

**Table 4:** Migration rates between populations estimated with BayesAss 3.0.

	Transition zones included		Transition zones excluded	
	Migration rate	95% CI	Migration rate	95% CI
eastern ⇨ recolonized	0.090	0.061–0.120	0.059	0.029–0.088
Carpathian ⇨ recolonized	0.007	0.000*–0.015	0.002	0.000*–0.006
recolonized - nonmigrants	0.903	0.873–0.932	0.940	0.910–0.969
recolonized ⇨ eastern	0.070	0.040–0.101	0.019	0.000*–0.039
Carpathian ⇨ eastern	0.008	0.000*–0.020	0.009	0.000*–0.024
eastern - nonmigrants	0.922	0.891–0.953	0.972	0.948–0.996
recolonized ⇨ Carpathian	0.015	0.000*–0.037	0.028	0.003–0.053
eastern ⇨ Carpathian	0.036	0.002–0.070	0.019	0.000*–0.045
Carpathian - nonmigrants	0.949	0.916–0.982	0.953	0.921–0.986

Recolonized – grouped regions WPL I, WPL II, NWPL, VRV and CentrPL, eastern – grouped regions NEPL I, NEPL II, LT, BY-UA and SEPL. 0.000* indicates negative value of lower 95% confidence interval. Transition zones excluded - analyses not including CentrPL and NEPL I regions.

## Discussion

### Genetic diversity of wolf population in newly recolonized areas

Microsatellite diversity measures such as allelic richness were generally lower in recently colonized regions west of the Vistula river compared to Baltic and Carpathian wolf populations (*sensu* Chapron *et al*.^15^). However, we observed important regional differences. In the north-western Poland (regions NWPL and WPL II) diversity was relatively high compared to south-western regions (mostly WPL I, but also VRV). These results correlate well with mtDNA diversity patterns - in northern regions haplotypes w1 and w2 were present at similar frequency, and a third haplotype, w6, was also detected, while the south-west was strongly dominated by only one (w1) haplotype. This can be attributed to different rate of immigration from the east, as more individuals assigned to the eastern populations in clustering analyses were detected in the north-west.

Despite the lower diversity in recolonized regions, we detected no evidence of inbreeding, as F_IS_ values were generally close to 0. On the contrary, in the two regions with the lowest microsatellite allelic richness (WPL I and VRV) the observed heterozygosities were higher than expected and F_IS_ values were slightly negative. A similar pattern was found when the diversity of clusters identified in STRUCTURE was analyzed: the western clusters at both partitioning levels (K = 3 and K = 5) had the lowest A_R_, but H_O_ exceeded H_E_ and F_IS_ was negative (Table 5). These results are in accordance with previous research suggesting inbreeding avoidance in canids^38^ and particularly in wolves^39–41^. Moreover, western Poland is generally well connected by ecological corridors^42^, enabling high gene flow rate within this region. This is reflected by the lack of detectable internal substructure within the western genetic cluster.

**Table 5:** Microsatellite summary statistics for clusters identified in STRUCUTRE analyses.

	N	Na	A_R_	H_O_	H_E_	uH_E_	F_IS_	PA
**clusters at K** = **3**								
1 - western	145	5.154	4.816	0.598	0.587	0.589	−0.015	0
2 - eastern	166	7.538	7.103	0.699	0.719	0.721	0.030	15
3 - Carpathian	69	6.615	6.604	0.670	0.677	0.682	0.018	7
**clusters at K = 5**								
1 - western	113	4.692	4.223	0.605	0.577	0.579	−0.044	0
2 - north-eastern	61	7.077	6.578	0.744	0.731	0.737	−0.010	11
3 - Carpathian	62	6.308	5.926	0.660	0.667	0.673	0.019	7
4 - northern	44	5.154	4.820	0.579	0.590	0.597	0.030	2
5 - south-eastern	35	5.231	5.183	0.687	0.659	0.669	−0.028	3

Observed heterozygosity values were much higher than those reported by Czarnomska and coworkers^16^, who detected also high values of an inbred coefficient in Polish wolves. These discrepancies are quite surprising, given that sets of microsatellite markers used here and in the aforementioned work largely overlapped. However, the dataset used by Czarnomska and coworkers had relatively high level of allele dropout (up to 36% missing data in some markers), while our procedures of isolation and amplification of DNA from non-invasive samples allowed to reduce the average dropout rate to below 4%. Thus, the observed heterozygosity and inbred coefficient values recorded in this study better represent the actual diversity of studied populations. Expected heterozygosity values, which are less biased by large dropout rates, in the study of Czarnomska and coworkers are similar to those reported here.

### Genetic clustering results support designation of the central european wolf population

The clustering solutions of different analyses are generally compatible, showing relatively clear separation of Carpathian, lowland eastern and western wolf populations. The only discrepancies concern the substructure inside the eastern cluster: the northern cluster identified in STRUCTURE was not recognized in spatial GENELAND analysis, while DAPC results suggested that it may be an intermediary cluster between the western and north-eastern clusters. However, designation of a separate south-eastern genetic group was well supported, as assignments of STRUCTURE at K = 5 and GENELAND at K = 4 were concordant, and both the SEPL region and the south-eastern cluster were separated from other groups in DAPC (by DA3 in general analyses or DA2 when Carpathians were excluded).

Surprisingly, in STRUCTURE analyses at K = 2 the eastern lowland wolves clustered with Carpathians, indicating very strong differentiation of the western cluster. However, it did not have any private alleles, as all alleles occurring in this cluster were present in the gene pool of the eastern cluster (at the K = 3 level of partitioning). This is in agreement with the results of an earlier study^16^, which suggested a north-eastern origin of the few first wolf family groups already established west of the Vistula river. However, allele frequencies were strongly altered, with some alleles (e.g. allele 157 in locus FH2137) with relatively low frequency in the east but being dominant in the west, indicating founder-flush event^43^ and allele surfing^26^. Noteworthy, the western regions were much more differentiated from the Carpathian wolf population than the eastern lowland regions.

Further genomic, ecological and behavioral research is necessary to determine if the observed genetic distinctiveness of western Polish wolves is just an effect of stochastic processes during range expansion and demographic growth, related for example to higher spreading potential of alleles “surfing” at the expansion front, as described in several empirical studies^44–46^ or whether it is connected with functional adaptations to new habitats (e.g. higher anthropopressure tolerance). In several recent studies, also a combination of demographic and adaptive processes in expanding populations was described^47^, related for example to the “Olympic Village Effect”^48^. Despite this ambiguity, there is no doubt that the wolf population newly established west of the Vistula river genetically differs from wolves inhabiting Lithuania, Belarus and easternmost parts of Poland and therefore cannot be considered as a part of the Baltic population. Our results are supported also by a recent study suggesting that the wolf population inhabiting eastern Germany (adjacent to western Poland) is genetically distinct from the Baltic wolves^49^. Thus, the current status of a discrete conservation unit (the Central European Lowland population) for the western Polish and German wolves seems to be fully justified.

### Central Poland - a contact zone and a melting pot of distinct wolf populations

Central Poland is characterized by high landscape fragmentation^50,51^, with only one relatively large forest tract indicated as a good wolf habitat by the suitability model^25^. Although wolves have been recorded there at least since the first decade of 21^st^ century^52^, their genetic origin have not been studied before. We found that this region is characterized by high wolf genetic diversity at both mitochondrial and nuclear DNA levels. It was the only region studied by us where all four main mtDNA haplotypes were present. Concordantly, we found there wolves representing each of the five clusters identified in STRUCTURE analyses, as well as several admixed individuals. According to GENELAND analyses, it was the region where the delineated borders of all populations (both at K = 3 and K = 4) met. BayesAss analysis indicated high gene flow rate from both western and eastern regions to the CentrPL region, and lower, but non-negligable migration from the Carpathians. Thus, our results suggest that central Poland is a contact zone of Carpathian, lowland south-eastern (that, as indicated by high frequency of the w3 haplotype, may be of Pontic origin), north-eastern (Baltic) and western wolf genetic groups. It also seems to be an admixture hotspot, because we identified there wolf packs in which breeding individuals were assigned to different clusters. Further development of the wolf population in this area is particularly interesting and worth further genetic and ecological research.

### Recolonization of mountainous areas by lowland wolves

Our dataset included six wolves from all three resident family groups which occurred in the Sudetes Mts (south-western Poland), recolonized by this species after over 200 years of absence^53^. All of those individuals had a haplogroup 1 mtDNA haplotypes (w1 – five wolves, and w2 – one wolf) typical for Central/Eastern European lowlands^29^. Concordantly, they were assigned to lowland populations in all Bayesian clustering analyses and DAPC. This finding is particularly significant as one of the most likely explanations of the differentiation between Polish lowland and Carpathian wolves was the natal habitat biased dispersal hypothesis^16,21^. Areas in the Sudetes Mts occupied by wolves are separated from the western edge of the Carpathian Mts by less than 200 km and their landscape characteristics much more resemble Carpathians than lowlands in western Poland^54,55^. Even though we are aware that our results must be treated with caution as the recolonization of the Sudetes Mts is very recent and the dataset includes small number of wolves, they are supported by another study which found that the Czech part of this mountain range was settled by wolves assigned to the lowland population^20^. Thus, the case of Sudetes highlights the great ecological plasticity of the expanding Central European wolf population. This also highlights the possible importance of anthropogenic factors, as the eastern edge of the Sudetes Mts is separated from the Carpathians by a relatively narrow, but densely populated and urbanized Moravian Gate^42,55^.

On the other hand, we also detected likely dispersals of Carpathian wolves to lowlands. One pack in the CentrPL region was concordantly classified as of Carpathian origin in mtDNA, STRUCTURE, GENELAND and DAPC analyses. A pack of wolves bearing the Carpathian w6 haplotype was detected also in the NWPL region, but microsatellite data revealed their north-eastern origin, suggesting a past gene flow between the Carpathian and Baltic population. This was supported by the Bayesian clustering analysis of the Lithuanian wolf population, where several individuals bore traces of admixture with the Carpathian population. Admixed individuals were detected also in Belarus, Ukraine and in small forest patches in the region NEPL II, but not in the main forest tracts in lowland Poland, including the SEPL region adjacent to the Carpathian Mountains. It can be explained as a high density blocking effect^27,56^: bigger Polish forests are saturated with wolf packs, which leaves little space for Carpathian dispersers, forcing their long-distance movement abroad to regions that are less saturated, with vacant territories due to heavy harvest of wolves in Ukraine, Belarus and Lithuania.

### Implications of genetic clustering results for future management and conservation plans

One of the most important steps in conservation planning for populations of wild-living species is the recognition of their intraspecific diversity followed by delineation of adequate management units (MUs)^57^. Identification of MUs is primarily justified by the amount of genetic divergence at which populations become demographically independent^58^. However, to avoid management failures, identification of MUs should consider not only biological or geographical features, but also social and political factors^59^.

There are several MUs identified for European wolves^60,61^, for which specific key actions has already been proposed^62^. Originally delineation of those MUs (called populations) was based mainly on data on wolf distribution, geographic features (e.g. existence of barriers), habitat quality, dispersal abilities and different management conditions. However, their authors called for further research on population genetics to allow revision of the population structuring^60^.

Subpopulations delineated by our study meet the criteria of MUs defined by Moritz^63^, who described them as “populations with significant divergence of allele frequencies at nuclear or mitochondrial loci, regardless of the phylogenetic distinctiveness of the alleles”. In this study we confirmed separation of Carpathian, Baltic and Central European wolf populations, and supported the proposal of Linnell and coworkers^60^ to treat them as discrete MUs. Moreover, we suggest to consider an additional MU (South-Eastern European wolf population) located in the south-eastern Poland, which we found to be genetically different from all other subpopulations (Suppl. Fig. S6). This is in concordance with earlier findings^16,21^, which described a discrete subpopulation situated latitudinally between Carpathian and Baltic regions and extending eastward, beyond the Polish state border, through lowlands of Ukraine and Belarus and further to the Pontic steppe. This genetic differentiation is connected with specific environmental variables^21^ and prey preferences^64^. Thus, we propose an update of MUs for wolves in Central Europe, taking under consideration all former studies together with the results of our analysis (Suppl. Fig. S7). We are aware, that the exact borders between MUs should be treated with caution due to the exchange of individuals, which is the most intense at their edges. Nonetheless, we believe that our suggestion will be useful for future population-level management of wolves in Central Europe and will fuel wider international co-operation for conservation of this carnivore.

## Conclusions

The most important finding of this study is the genetic distinctiveness of the wolf population inhabiting western Poland from neighboring populations. Thus, we conclude that it should be treated as a separate conservation unit. Genetic diversity of the western Polish population is lower, when compared to the Baltic population, due to the founder effect and limited east-west gene flow. However, the within-population gene flow rate must be high, as indicated by the lack of internal substructure, relatively high observed heterozygosity and no detectable inbreeding. The revealed patterns of recolonization (e.g. expansion of lowland wolves to mountainous areas) confirm that the detected genetic structure of wolf population in Central Europe is shaped more by the recent demographic history than by ecological factors.

## Methods

### Study area

Although our study focused mainly on the Polish wolf population, we also analyzed samples collected in Lithuania, Belarus, Slovakia and eastern Czech Republic. Thus the total study area stretches between 48°5′–56°4′N and 14°1′–30°5′E. The landscape of this part of Europe was profoundly shaped by Pleistocene glaciations, and consists mainly of lowlands, up to 200 m above sea level, with some frontal and moraine hills, which in northern parts are accompanied by lakes. Southern part of the study area encompasses two mountain ranges – Sudetes Mts. (max. altitude 1,603 m a.s.l.) and Carpathian Mts. (max. 2,655 m a.s.l.).

The area has a transitional continental–Atlantic climate, with mean temperatures from −1.1 to 0.6 °C in January, and from 18.1 to 19.5 °C in July. Mean precipitation ranges from 500 to 800 mm. The majority of the area is an agricultural land, however the forest cover is from 30% in Poland to almost 40% in Belarus. Forests are dominated by managed stands of Scots pine *Pinus sylvestris* and Norway spruce *Picea abies*, but in southern and western parts there are larger patches of mixed and deciduous woods with prevalence of beech (*Fagus sylvatica*), birches (*Betula* sp.), hornbeam (*Carpinus betulus*), oaks (*Quercus* sp.), alders (*Alnus* sp.) and poplars (*Populus* sp.).

In central Europe the wolf co-occur with two other large carnivores – more widespread Eurasian lynx (*Lynx lynx*) and brown bear (*Ursus arctos*) which is restricted to the Carpathian Mts. and lowlands in Baltic states^15^. Moreover, the region is characterized by abundant populations of wild ungulates being main wolf prey^64^ – red deer (*Cervus elaphus*), roe deer (*Capreolus capreolus*) and wild boar (*Sus scrofa*), with less numerous moose (*Alces alces*), locally reintroduced European bison (*Bison bonasus*), and chamois (*Rupicapra rupicapra*) restricted to mountains. There are also isolated populations of alien species introduced for hunting purposes, such as fallow deer (*Dama dama*), sika deer (*C. nippon*) and mouflon (*Ovis musimon*), as well as a large population of the Eurasian beaver (*Castor fiber*).

The area has a clear north-east– south-west gradient of human population density, from around 45 people/1km^2^ in Lithuania and Belarus up to 110–130 people/1km^2^ in Poland, Slovakia and Czech Republic. Similar patterns are observed in regard to urbanization and density of transport infrastructure^65,66^.

### Sampling strategy

Our main goal was to obtain a dense, representative sampling of previously unstudied areas west of the Vistula river that were recolonized by wolves over the last decade^14^. As reference, we gathered DNA from wolves inhabiting main forest tracts in eastern Poland and in western Carpathian Mountains. Additionally, we collected samples from wolf family groups that recently have established in some relatively small forest complexes in north-eastern Poland (NEPL_I and NEPL_II regions, see Fig. 1) that are particularly interesting as those habitats were classified as suboptimal^25^, as well as packs inhabiting south-eastern Poland (SEPL), a region that was found to host a distinct wolf subpopulation based on previous mtDNA analysis^16^. To compare the genetic structure of Polish wolf population with adjacent regions, we analyzed also samples gathered in Lithuania, Belarus, Ukraine (Chernobyl zone), Slovakia and eastern Czech Republic.

Non-invasive samples N = 2110 (mainly scats – N = 1792 but also urine – N = 139, hair – N = 139, blood from estrus – N = 29 and swabs from wolf kills – N = 11) were collected from 2011 to 2018 by authors and trained volunteers all year round, during long-distance wolf tracking on forest roads, tourist trails and around known wolf dens and rendezvous sites (see^14^ for more details). We also gathered tissue and hair samples from wolves killed in traffic accidents (N = 97), illegally shot or snared (N = 29) or found dead due to diseases and other natural causes (N = 16). Additionally, we analyzed blood and hair samples of animals injured in traffic accidents or by poachers (N = 9). Lastly, we analyzed tissue samples from wolves hunted legally in Lithuania (N = 63) and Slovakia (N = 23). No animals were specifically killed or captured for this study. Distribution of sampling locations is shown on Fig. 1. Samples were divided into groups corresponding to geographic regions, which borders were based on possible dispersal barriers (e.g. the Vistula river), the results of previous research concerning genetic structure of wolf populations in Central Europe^16,20,21^ or, in case of regions unstudied before, dynamics of their recolonization by wolves^14,17^. As a presence of dog-specific alleles can potentially interfere in downstream population structure analyses, we aimed at the elimination of all putative wolf-dog hybrids from our dataset. Thus, additionally, 50 dog samples (hairs, blood or tissues) were collected from veterinarians and private owners and from individuals killed by vehicles and used as a reference group to detect possible wolf-dog hybrids. Dog owners granted us permission for the use of these samples in research. Details concerning this procedure can be found in Supplementary Methods.

Scat and tissue samples were fixed with 96% ethanol and stored at+ 4 °C, while hairs, swabs and blood collected on FTA cards (Whatman) were kept at room temperature in dry paper envelopes containing desiccant. Urine samples were mixed with 2 volumes of 96% ethanol and sodium acetate (100 mM final concentration) and kept at −20 °C.

### Laboratory analyses

DNA isolation from non-invasive samples was performed in a separate cleanroom to avoid contamination. DNA from scats was isolated either with QIAamp DNA Stool Mini Kit (Qiagen) or Exgene™ Stool DNA Mini kit (GeneAll Biotechnology), while for hairs, swabs and FTA cards we used Exgene™ Genomic DNA Micro kit (GeneAll Biotechnology) or QIAamp DNA Investigator Kit (Qiagen). DNA from tissues and precipitated urine samples was isolated with Exgene™ Tissue SV kit (GeneAll Biotechnology).

To analyze the matrilineal genealogy we amplified the left variable domain of the mitochondrial control region using primers L15995^67^ and H16498^68^. This domain contains a 230 bp fragment rich in parsimony-informative sites, enabling its use for a range-wide wolf haplotype classification^20,21^. PCR was performed in 15 μl reaction volume containing 1X DreamTaq Green PCR Master Mix (ThermoScientific), primers at concentration of 0.33 μM each, 0.17 μg/μl BSA and 3 μl DNA extract (typically 5–20 ng DNA). The PCR reaction started with an initial denaturation at 95 °C (3 min) followed by 35 cycles of 94 °C (15 s), 54 °C (30 s) and 72 °C (60 s), and a final elongation of 72 °C (10 min). PCR products were purified using exonuclease I and FastAP alkaline phosphatase (ThermoScientific) and sequenced on ABI3730/xl Genetic Analyzer (Applied Biosystems). Chromatograms were analyzed and edited in FinchTV 1.4 and compared to sequences deposited in the NCBI database.

To infer population structure we analyzed 13 polymorphic microsatellite loci: FH2001, FH2010, FH2017, FH2054, FH2087L, FH2088, FH2096, FH2137, FH2140, FH2161^69^, vWF^70^, PEZ17^71^ and CPH5^72^. Additionally, DBX intron 6 and DBY intron 7^73^ were used as sex markers. Loci were amplified in three 10 μl multiplex reactions, each containing 5 μl Multiplex PCR Master Mix (Qiagen), 0.25 μg/μl BSA, primers at concentration 0.2 μM each and 4.2 ul DNA extract. PCR was started with initial denaturation (95 °C, 15 min) followed by 4 cycles of 94 °C (30 s), 60 °C (90 s) and 72 °C (60 s); another 5 cycles of 94 °C (30 s), 58 °C (90 s) and 72 °C (60 s), 5 cycles of 94 °C (30 s), 56 °C (90 s) and 72 °C (60 s); another 5 cycles of 94 °C (30 s), 54 °C (90 s) and 72 °C (60 s), 25 cycles of 94 °C (30 s), 50 °C (90 s) and 72 °C (60 s), and a final elongation 30 min at 72 °C. PCR products were separated by electrophoresis using an ABI3730/xl Genetic Analyzer with the internal size standard GS600 LIZ™ (Applied Biosystems) and allele sizes were binned using PeakScanner 1.0 software. To avoid allele dropout and genotyping errors we utilized a multitube amplification approach^74^. However, as the reliability of a genotype could be predicted based on sample quality^75^, the number of multitube replicates varied depending on amplification quality. Initially each non-invasive DNA sample was amplified twice. At this stage low quality samples (>35% missing data) were discarded and good quality genotypes (no missing data, ≤1 mismatch between replicates) were accepted. For the remaining samples two additional PCR repetitions were performed and consensus genotypes were reconstructed from tetraplicates.

### Population structure analyses

Multilocus genotypes were compared and collapsed in GenAlex^76^. We accepted one mismatching allele to consider genotypes as belonging to the same individual. Evaluation of allele frequencies, expected and observed heterozygosity, identification of private alleles, calculation of R_ST_ values and estimation of probability of identity of multilocus genotypes between unrelated individuals (P _ID_) and siblings (P _ID-sib_) were also done in GenAlex. Tests for the presence of false and null alleles were done in Microchecker^77^. Rarefied allelic richness (Ar), inbreeding coefficient (F_IS_) and fixation index (F_ST_) were estimated using FSTAT^78^.

Closely related individuals were identified by a combination of methods: 1) estimation of relatedness between pairs of individuals using the estimator of Lynch & Ritland^79^ implemented in GeneAlex, 2) parentage and sibship analysis in software Cervus 3.0^80^ and Colony 2^81^ and 3) manually by direct genotypes comparison.

Genetic clusters were detected using two Bayesian approaches: the non-spatial model implemented in STRUCTURE 2.3.4^82^ and spatial clustering algorithms used by GENELAND 4.0.8. In STRUCTURE, 10^6^ MCMC iterations were run following initial burn-in of 10^5^ steps using the admixture model with correlated allele frequencies and no prior population information. As STRUCTURE runs are stochastic and may produce different outcomes for replicate runs, even when the same choice of model and parameters is used, ten independent repetitions were run for each K value (varying from 1 to 12). The Evanno method^30^ applied in STRUCTURE HARVESTER^83^ was used to infer the most likely number of clusters. Separate STRUCTURE runs were aligned and merged in CLUMPAK^84^ application using implemented there DISTRUCT^85^ and CLUMPP LargeKGreedy^86^ algorithms, respectively. As one-step STRUCTURE analysis may not always reveal substructures in datasets containing highly divergent populations, we utilized also a multi-step approach that has been proven useful in identifying hierarchical population structure^87,88^. For each of the main clusters identified in the first step, a separate analysis was rerun using the same parameters. In case of GENELAND, we performed analyses using both the uncorrelated and correlated allele frequency models, as the latter may be more powerful at detecting subtle structure, but on the other hand is more sensitive to deviations from model assumptions and thus more prone to algorithm instabilities than the uncorrelated model^89,90^. Each model was run ten times with 5 × 10^5^ MCMC iterations, a thinning of 100, a maximum rate of Poisson process fixed to 100, a spatial coordinates uncertainty fixed to 0.1°, a maximum number of nuclei in the Poisson–Voronoi tessellation fixed to 500 and a range of tested K values from 1 to 15. Posterior probabilities of population membership for each pixel and individual were calculated with a burn-in of 1000. In case of inconsistency between multiple runs results, best runs were selected based on mean probability density values.

Additionally, we analyzed microsatellite data using a multivariate method DAPC (discriminant analysis of principal components)^32^ implemented in the R package *adegenet*^91^. DAPC is a model-independent method, and therefore free of population genetics assumptions such as Hardy-Weinberg equilibrium. Separate analyses were performed for the whole dataset divided a priori into groups corresponding to geographic regions from Fig. 1, reduced datasets and for clusters identified in STRUCTURE. The optimum number of retained principal components was determined using the cross validation method.

Contemporary migration rates between regions and identified genetic groups were estimated using BayesAss 3.0^36^. Ten independent runs were performed, each for 10^7^ iterations with a burn-in of 10^6^ and sampling every 100 iterations. Rough 95% confidence intervals were calculated as mean ± 1.96 × SD, as recommended by the software’s manual.

## Supplementary information

Supporting Information

## Data Availability

Upon acceptance of the manuscript, wolf microsatellite genotypes used in the final analyses will be deposited at Dryad Digital Repository.
