# Supplementary material for: Dynamic range expansion leads to establishment of a new, genetically distinct wolf population in Central Europe
Source: Sci Rep. 2019 Dec 12;9:19003. doi: 10.1038/s41598-019-55273-w (PMC6908625; doi:10.1038/s41598-019-55273-w)
Supplement: Supplementary file 1 — Supporting Information [file 41598_2019_55273_MOESM1_ESM.pdf]

Supporting Information for

Dynamic range expansion leads to establishment of a new, genetically distinct wolf population in Central Europe

Maciej Szewczyk[1,2,3], Sabina Nowak[2], Natalia Niedźwiecka[2], Pavel Hulva[4,5], Renata Špinkytė-Bačkaitienė[6], Klára Demjanovičová[5], Barbora Černá Bolfíková[7], Vladimír Antal[8], Viktar Fenchuk[9], Michał Figura[2], Patrycja Tomczak[2,10], Przemysław Stachyra[11], Kinga M. Stępniak[1,2], Tomasz Zwijacz-Kozica[12] & Robert W. Mysłajek[1,*]

[1] Institute of Genetics and Biotechnology, Faculty of Biology, University of Warsaw, Pawińskiego 5a, 02-106 Warsaw, Poland
[2] Association for Nature „Wolf", Twardorzeczka, Cynkowa 4, 34-324 Lipowa, Poland
[3] Department of Vertebrate Ecology and Zoology, Faculty of Biology, University of Gdańsk, Wita Stwosza 59, 80-308 Gdańsk, Poland
[4] Faculty of Science, Charles University in Prague, Viničná 7, 128 43 Prague, Czech Republic
[5] Faculty of Science, University of Ostrava, Chittussiho 10, 170 00 Ostrava, Czech Republic
[6] Vytautas Magnus University, K. Donelaičio 58, 44248 Kaunas, Lithuania
[7] Department of Animal Science and Food Processing, Faculty of Tropical AgriSciences, Czech University of Life Sciences Prague, Kamýcká 129, 165 00 Prague 6, Czech Republic
[8] State Nature Conservancy of Slovak Republic, Tajovského 28B, 974 01 Banská Bystrica, Slovakia
[9] APB-BirdLife Belarus, Engelsa 34A - 1, 220030 Minsk, Belarus
[10] Institute of Romance Studies, Faculty of Modern Languages and Literature, Adam Mickiewicz University in Poznań, Al. Niepodległości 4, 61-874 Poznań, Poland
[11] Roztocze National Park, Plażowa 2, 22-470 Zwierzyniec, Poland
[12] Tatra National Park, Kuźnice 1, 34-500 Zakopane, Poland

*Corresponding author: Robert W. Mysłajek, e-mail: robert.myslajek@igib.uw.edu.pl; phone: +48 604625228

TABLE OF CONTENTS

Title	Page
Fig. S1: Identification and exclusion of wolf-dog hybrids	2
Fig. S2: STRUCTURE results for the whole dataset (881 wolf individuals)	3
Fig. S3: Results of STRUCTURE clustering of reduced datasets	4
Fig. S4: Results of hierarchical STRUCTURE analysis	5
Fig. S5: Raw GENELAND results	6
Fig. S6: Results of DAPC analyses supporting distinctiveness of south-eastern cluster	7
Fig. S7: Proposed delineation of management units for wolves in Poland	8
Table S1: Microsatellite summary statistics per locus	9
Table S2: Migration rates estimated with BayesAss 3.0, assuming 4 populations	12
Table S3: Migration rates estimated with BayesAss 3.0, assuming 6 populations	13
Table S4: Migration rates between 11 regions estimated with BayesAss 3.0	14
Supplementary method: Identification of putative wolf-dog hybrids	16
References	17

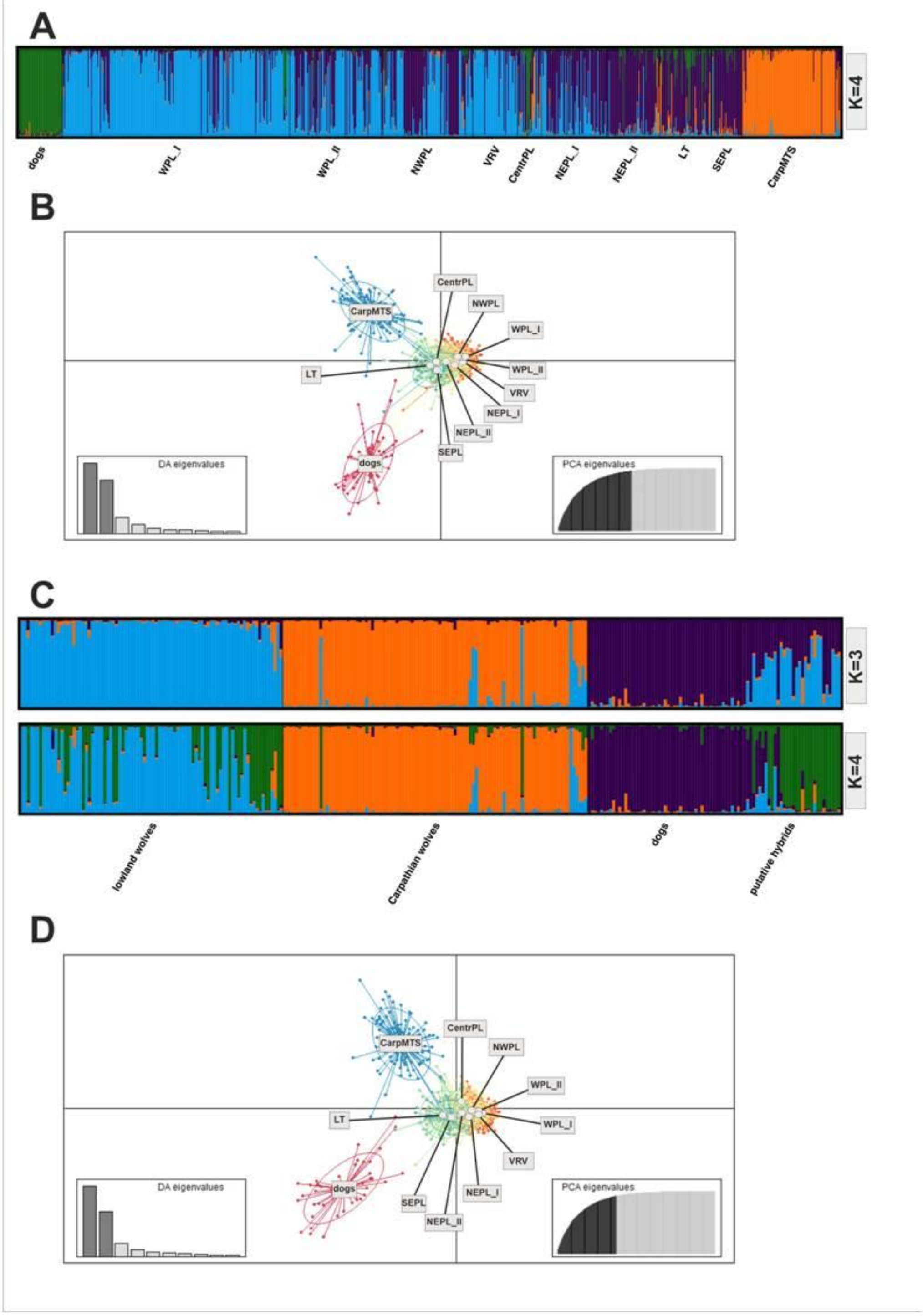

Fig. S1: Identification and exclusion of wolf-dog hybrids. Panels A-B – analysis of the whole dataset (894 putative wolves and 50 reference dogs) with STRUCTURE (A) and DAPC (B), panel C – analysis of putative hybrids identified in panel A against smaller dataset of reference wolves and dogs, D – DAPC analysis of the dataset after removal of 13 identified wolf-dog hybrids.

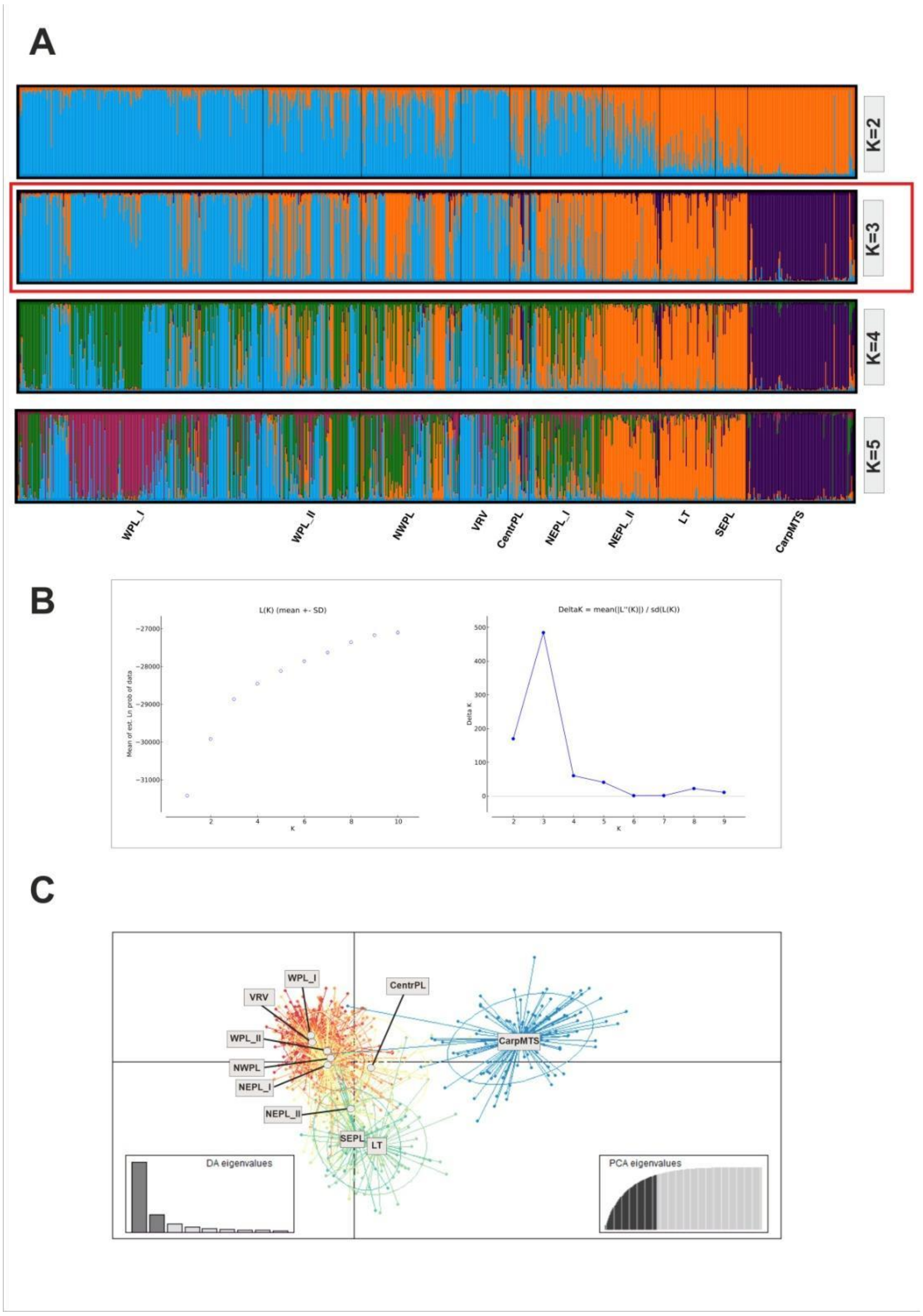

Fig. S2: Analysis of the whole dataset of 881 identified wolf individuals. Panel A – bar plots of STRUCTURE results at K range from 2 to 5. Best K value (as indicated by Evanno method) is indicated with red rectangle, panel B – plots representing analysis of STRUCTURE results for the whole tested range of K with the Evanno method, panel C – results of DAPC analysis.

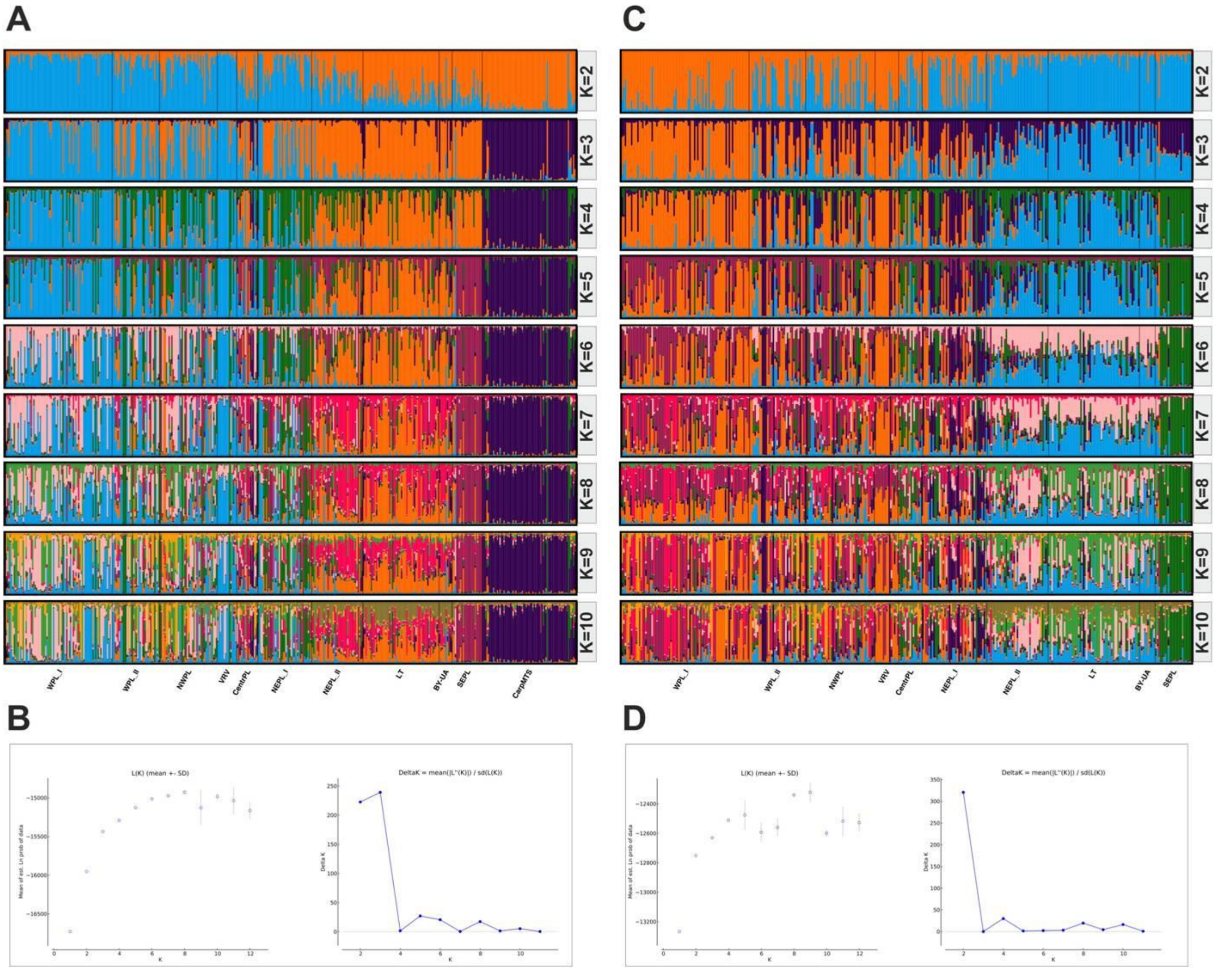

Fig. S3: Results of STRUCTURE clustering of reduced datasets. Panels A-B: analysis covering all geographic regions, panels C-D: analysis excluding Carpathians.

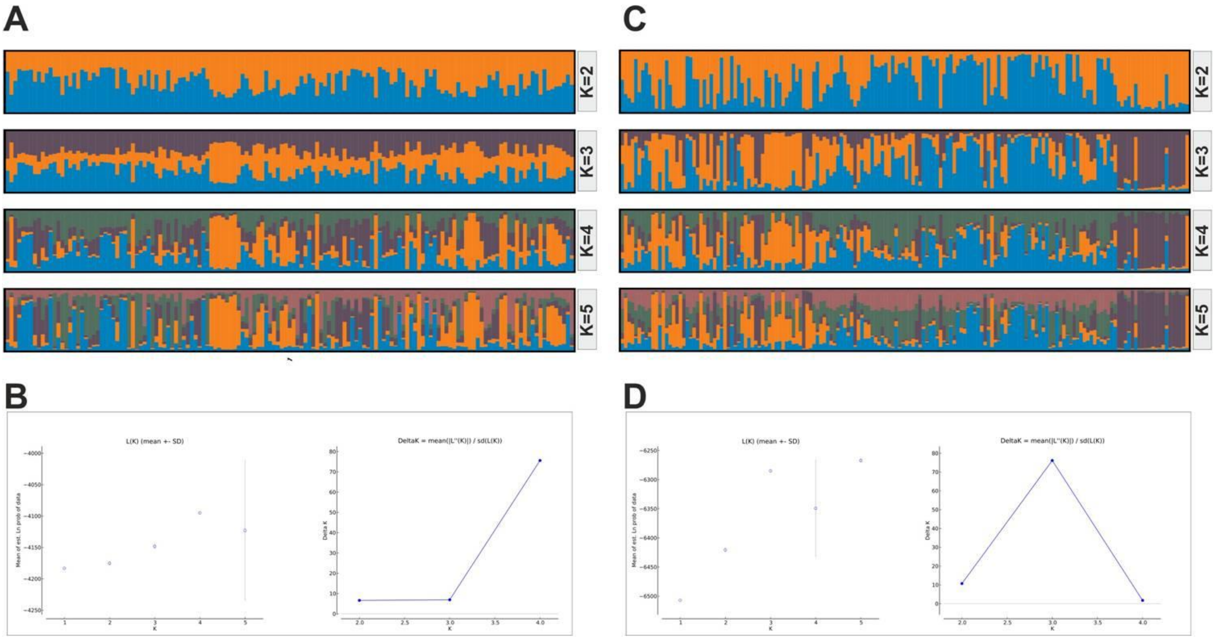

Fig. S4: Results of hierarchical STRUCTURE analysis of previously identified clusters: panels A-B: western cluster, panels C-D: eastern cluster.

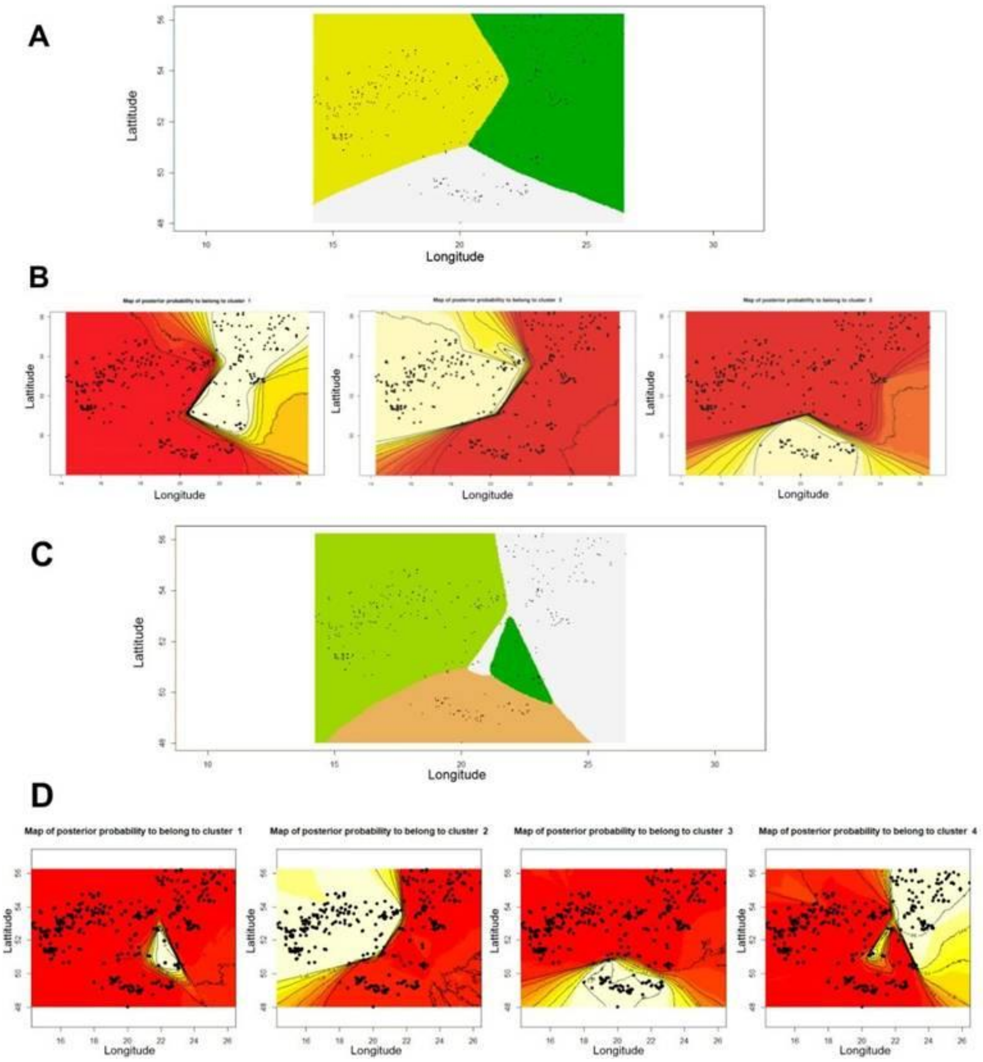

Fig. S5: Raw GENELAND results: identified clusters (panels A and C) and maps of posterior probabilities of cluster assignments (panels B and D) for analyses utilizing the uncorrelated allele frequency model at K=3 (A-B) or K=4 (C-D).

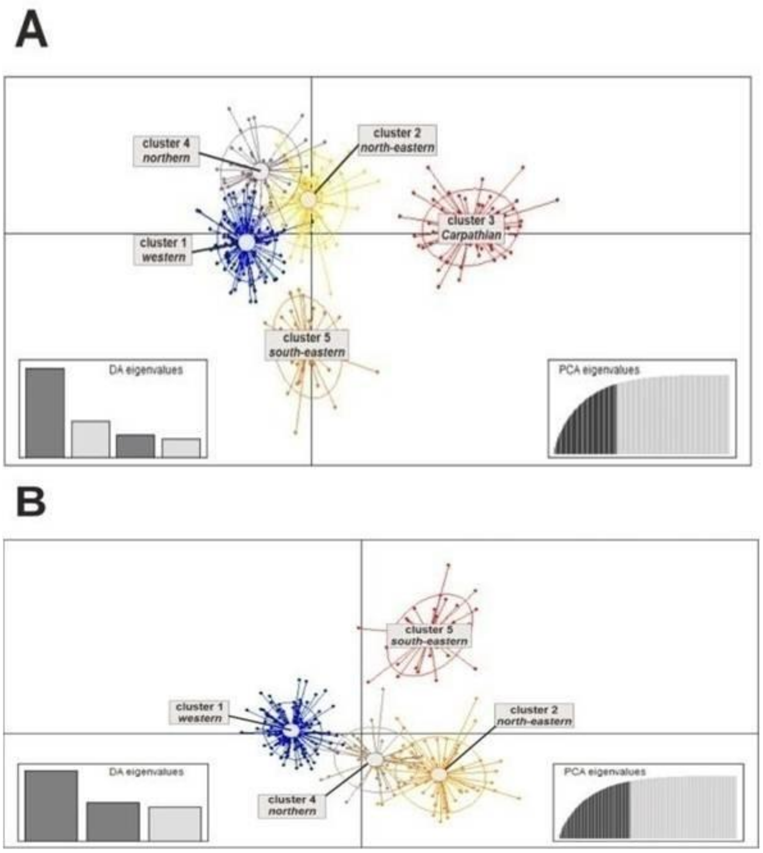

Fig. S6: Results of DAPC analyses supporting distinctiveness of south-eastern cluster. panel A – the same analysis as presented on Fig. 5 D, but DA3 instead of DA2 is represented on y-axis; panel B – additional analysis not including the Carpathian cluster.

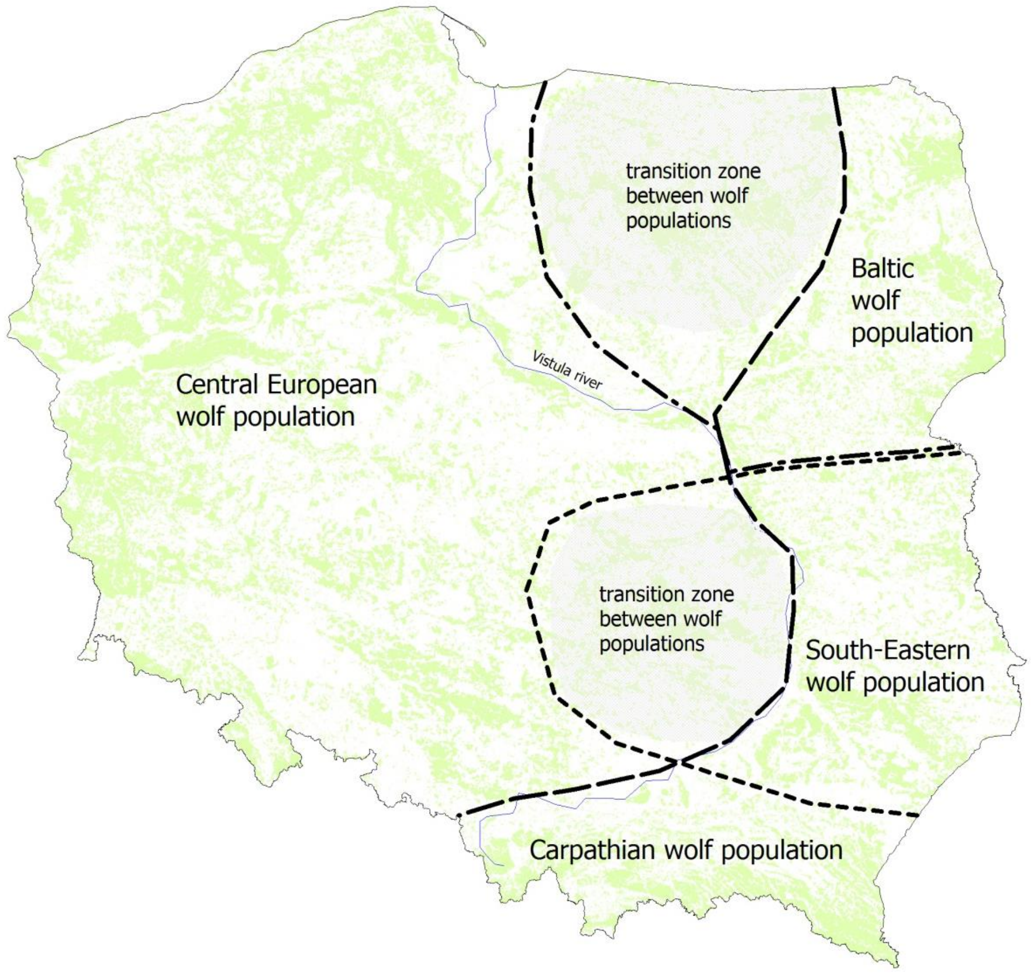

Fig. S7: Proposed delineation of management units (MUs) for wolves in Poland.

Table S1: Per locus microsatellite summary statistics for the 10 predefined geographic regions

Region	Locus	N	Na	A_R	H_O	H_E	uH_E	F_{IS}
WPL I	FH2001	249	4	3.951	0.695	0.682	0.683	-0.017
	FH2087	247	7	4.849	0.676	0.660	0.662	-0.022
	FH2096	227	4	3.653	0.476	0.473	0.475	-0.003
	PEZ17	252	5	3.240	0.532	0.476	0.477	-0.115
	FH2010	244	5	4.940	0.721	0.686	0.687	-0.050
	FH2017	212	4	3.962	0.524	0.509	0.510	-0.026
	FH2137	253	9	3.706	0.229	0.225	0.225	-0.017
	vWF	254	6	3.604	0.669	0.644	0.645	-0.038
	FH2088	250	6	5.680	0.720	0.757	0.758	0.051
	FH2140	246	4	3.510	0.602	0.614	0.616	0.023
	FH2054	251	6	5.216	0.530	0.645	0.646	0.180
	FH2161	244	9	6.908	0.725	0.718	0.720	-0.008
	CPH5	255	4	3.080	0.667	0.608	0.609	-0.094
WPL II	FH2001	96	4	3.525	0.625	0.668	0.672	0.070
	FH2087	97	6	4.582	0.753	0.704	0.708	-0.064
	FH2096	100	4	3.966	0.590	0.576	0.579	-0.019
	PEZ17	99	4	3.212	0.525	0.631	0.634	0.172
	FH2010	95	6	4.606	0.737	0.687	0.690	-0.068
	FH2017	85	4	3.973	0.482	0.534	0.537	0.103
	FH2137	103	7	5.390	0.447	0.430	0.432	-0.034
	vWF	97	6	4.722	0.691	0.685	0.688	-0.004
	FH2088	103	6	5.031	0.738	0.685	0.688	-0.073
	FH2140	102	5	4.306	0.686	0.642	0.645	-0.065
	FH2054	94	7	5.789	0.787	0.788	0.792	0.006
	FH2161	95	8	7.474	0.684	0.765	0.769	0.110
	CPH5	101	3	2.999	0.634	0.597	0.600	-0.056
NWPL	FH2001	101	5	4.197	0.584	0.683	0.686	0.149
	FH2087	98	7	5.450	0.704	0.749	0.753	0.065
	FH2096	103	4	3.827	0.466	0.466	0.468	0.004
	PEZ17	98	4	3.214	0.724	0.588	0.591	-0.227
	FH2010	97	6	5.193	0.732	0.695	0.699	-0.048
	FH2017	94	4	3.954	0.468	0.573	0.576	0.189
	FH2137	96	9	6.100	0.385	0.410	0.412	0.066
	vWF	102	6	4.330	0.676	0.692	0.695	0.027
	FH2088	103	7	5.910	0.777	0.774	0.778	0.001
	FH2140	103	7	4.505	0.602	0.622	0.625	0.037
	FH2054	97	10	7.141	0.639	0.731	0.735	0.131
	FH2161	100	7	6.732	0.800	0.777	0.781	-0.025
	CPH5	103	4	3.843	0.592	0.610	0.613	0.034
VRV	FH2001	49	3	3.000	0.694	0.588	0.594	-0.170
	FH2087	48	5	4.430	0.708	0.666	0.673	-0.053
	FH2096	49	4	3.867	0.408	0.378	0.382	-0.069
	PEZ17	49	4	3.429	0.735	0.623	0.629	-0.170
	FH2010	46	5	4.704	0.587	0.657	0.664	0.117
	FH2017	48	4	3.433	0.604	0.475	0.480	-0.262
	FH2137	51	4	2.480	0.078	0.076	0.077	-0.018
	vWF	50	4	3.420	0.580	0.653	0.659	0.121

	FH2088	51	6	5.062	0.824	0.667	0.674	-0.224
	FH2140	51	4	3.656	0.588	0.656	0.662	0.113
	FH2054	50	7	5.944	0.680	0.700	0.707	0.039
	FH2161	49	7	6.375	0.735	0.737	0.745	0.014
	CPH5	51	3	3.000	0.765	0.628	0.635	-0.207
CentrPL	FH2001	21	5	5.000	0.762	0.703	0.720	-0.060
	FH2087	21	6	6.000	0.857	0.746	0.764	-0.125
	FH2096	22	4	3.955	0.455	0.439	0.449	-0.012
	PEZ17	22	5	4.955	0.591	0.717	0.734	0.198
	FH2010	22	5	5.000	0.682	0.688	0.704	0.032
	FH2017	21	4	4.000	0.619	0.643	0.659	0.061
	FH2137	22	5	4.953	0.682	0.536	0.549	-0.250
	vWF	22	5	4.909	0.636	0.679	0.695	0.086
	FH2088	22	6	5.955	0.682	0.735	0.752	0.095
	FH2140	22	6	5.952	0.682	0.683	0.699	0.025
	FH2054	22	8	7.951	0.818	0.778	0.796	-0.029
	FH2161	21	8	8.000	0.952	0.814	0.834	-0.146
	CPH5	22	3	3.000	0.591	0.569	0.582	-0.015
NEPL I	FH2001	71	5	4.628	0.634	0.670	0.675	0.062
	FH2087	68	7	5.611	0.662	0.786	0.792	0.165
	FH2096	74	4	3.958	0.446	0.442	0.445	-0.001
	PEZ17	68	6	4.729	0.544	0.642	0.647	0.159
	FH2010	72	6	5.326	0.681	0.648	0.653	-0.043
	FH2017	69	4	3.759	0.333	0.407	0.410	0.187
	FH2137	75	8	5.838	0.387	0.373	0.375	-0.030
	vWF	73	5	4.569	0.699	0.667	0.672	-0.041
	FH2088	74	6	5.655	0.716	0.727	0.732	0.022
	FH2140	73	4	3.628	0.548	0.590	0.594	0.079
	FH2054	73	9	5.873	0.603	0.678	0.682	0.117
	FH2161	71	9	7.993	0.775	0.752	0.758	-0.022
	CPH5	74	5	4.100	0.635	0.653	0.658	0.035
NEPL II	FH2001	57	5	4.738	0.614	0.679	0.685	0.104
	FH2087	55	7	6.337	0.764	0.789	0.796	0.042
	FH2096	60	4	3.929	0.667	0.608	0.613	-0.088
	PEZ17	59	4	3.832	0.644	0.626	0.632	-0.020
	FH2010	56	5	4.924	0.679	0.682	0.688	0.013
	FH2017	54	5	3.778	0.593	0.634	0.640	0.074
	FH2137	59	8	6.894	0.576	0.542	0.546	-0.055
	vWF	59	9	7.332	0.814	0.783	0.789	-0.031
	FH2088	59	6	5.324	0.729	0.712	0.718	-0.015
	FH2140	60	6	5.050	0.700	0.666	0.672	-0.042
	FH2054	55	8	6.799	0.709	0.770	0.777	0.088
	FH2161	57	8	7.440	0.825	0.810	0.817	-0.009
	CPH5	59	6	5.311	0.712	0.742	0.748	0.049
	FH2001	58	5	4.875	0.655	0.681	0.687	0.046
	FH2087	58	6	5.985	0.914	0.816	0.823	-0.111
	FH2096	58	4	3.936	0.586	0.584	0.589	0.005
	PEZ17	58	6	4.849	0.638	0.648	0.653	0.024
	FH2010	58	5	4.977	0.724	0.719	0.725	0.001
	FH2017	58	4	3.936	0.586	0.625	0.631	0.071

LT	FH2137	58	12	9.891	0.793	0.835	0.842	0.058
	vWF	58	9	7.557	0.828	0.821	0.829	0.001
	FH2088	58	7	6.179	0.776	0.770	0.777	0.001
	FH2140	58	8	6.562	0.741	0.730	0.737	-0.007
	FH2054	57	9	7.755	0.772	0.793	0.800	0.036
	FH2161	58	10	9.204	0.879	0.859	0.866	-0.015
	CPH5	58	4	3.744	0.707	0.650	0.655	-0.080
SEPL	FH2001	33	5	4.636	0.909	0.746	0.758	-0.204
	FH2087	31	4	3.997	0.516	0.599	0.609	0.154
	FH2096	34	3	2.948	0.735	0.528	0.536	-0.380
	PEZ17	34	6	5.217	0.706	0.667	0.676	-0.044
	FH2010	31	5	4.999	0.710	0.769	0.782	0.093
	FH2017	31	5	4.032	0.581	0.499	0.508	-0.146
	FH2137	33	5	4.507	0.576	0.626	0.635	0.095
	vWF	32	7	6.312	0.750	0.779	0.792	0.053
	FH2088	33	6	5.863	0.545	0.674	0.684	0.205
	FH2140	31	5	4.638	0.452	0.406	0.412	-0.097
	FH2054	33	7	6.827	0.788	0.784	0.796	0.011
	FH2161	32	8	7.161	0.813	0.792	0.805	-0.010
	CPH5	34	4	3.618	0.441	0.663	0.673	0.348
CarpMTS	FH2001	110	5	4.154	0.645	0.703	0.706	0.086
	FH2087	111	8	6.290	0.829	0.772	0.775	-0.069
	FH2096	111	4	3.701	0.523	0.498	0.500	-0.046
	PEZ17	108	7	6.100	0.815	0.777	0.781	-0.043
	FH2010	108	5	4.130	0.444	0.494	0.497	0.106
	FH2017	110	4	3.778	0.564	0.664	0.667	0.155
	FH2137	110	13	9.593	0.818	0.866	0.870	0.060
	vWF	111	9	6.411	0.586	0.655	0.658	0.110
	FH2088	109	7	5.342	0.734	0.685	0.688	-0.067
	FH2140	112	6	5.810	0.670	0.792	0.796	0.159
	FH2054	106	10	7.866	0.736	0.787	0.791	0.070
	FH2161	109	8	7.017	0.725	0.691	0.694	-0.045
	CPH5	109	4	3.884	0.358	0.387	0.389	0.081

Table S2: Migration rates between populations estimated with BayesAss 3.0, assuming 4 populations. Recolonized – grouped regions WPL I, WPL II, NWPL, VRV and CentrPL, north-eastern – grouped regions NEPL I, NEPL II, LT and BY-UA, south-eastern – SEPL region. 0.000* indicates negative value of lower 95% confidence interval. Transition zones excluded - analyses not including CentrPL and NEPL I regions.

	transition zones included		transition zones excluded	
	**migration rate**	**95% CI**	**migration rate**	**95% CI**
north-eastern ➪ recolonized	0.076	0.045 - 0.108	0.052	0.021 - 0.083
south-eastern ➪ recolonized	0.014	0.002 - 0.026	0.006	0.000* - 0.013
Carpathian ➪ recolonized	0.007	0.000 - 0.015	0.002	0.000* - 0.006
recolonized - nonmigrants	0.902	0.869 - 0.935	0.940	0.909 - 0.971
recolonized ➪ north-eastern	0.083	0.044 - 0.122	0.021	0.000* - 0.050
south-eastern ➪ north-eastern	0.029	0.004 - 0.054	0.022	0.000* - 0.047
Carpathian ➪ north-eastern	0.013	0.000* - 0.029	0.011	0.000* - 0.027
north-eastern - nonmigrants	0.875	0.832 - 0.918	0.947	0.904 - 0.990
recolonized ➪ south-eastern	0.038	0.000* - 0.084	0.040	0.000* - 0.088
north-eastern ➪ south-eastern	0.057	0.001 - 0.114	0.059	0.000* - 0.119
Carpathian ➪ south-eastern	0.015	0.000* - 0.043	0.015	0.000* - 0.042
south-eastern - nonmigrants	0.890	0.821 - 0.959	0.887	0.814 - 0.959
recolonized ➪ Carpathian	0.011	0.000* - 0.031	0.027	0.002 - 0.051
north-eastern ➪ Carpathian	0.039	0.005 - 0.072	0.016	0.000* - 0.040
south-eastern ➪ Carpathian	0.006	0.000* - 0.016	0.006	0.000* - 0.018
Carpathian - nonmigrants	0.945	0.912 - 0.978	0.951	0.918 - 0.984

Table S3: Migration rates between populations estimated with BayesAss 3.0, assuming 6 populations. Putative transition zones (CentrPL and NEPL I) were treated as separate populations. Recolonized – grouped regions WPL I, WPL II, NWPL and VRV, north-eastern – grouped regions NEPL II, LT and BY-UA, south-eastern – SEPL region. 0.000* indicates negative value of lower 95% confidence interval.

	**migration rate**	**95% CI**
CentrPL ⇨ recolonized	0.003	0.000* - 0.009
NEPL_I ⇨ recolonized	0.084	0.055 - 0.113
north-eastern ⇨ recolonized	0.019	0.001 - 0.038
south-eastern ⇨ recolonized	0.004	0.000* - 0.011
Carpathian ⇨ recolonized	0.002	0.000* - 0.006
recolonized - nonmigrants	0.887	0.857 - 0.918
recolonized ⇨ Centr_PL	0.178	0.101 - 0.256
NEPL_I ⇨ CentrPL	0.033	0.000* - 0.079
north-eastern ⇨ CentrPL	0.034	0.000* - 0.077
south-eastern ⇨ CentrPL	0.048	0.000* - 0.102
Carpathian ⇨ CentrPL	0.028	0.000* - 0.071
CentrPL - nonmigrants	0.680	0.655 - 0.705
recolonized ⇨ NEPL_I	0.182	0.121 - 0.243
CentrPL ⇨ NEPL_I	0.007	0.000* - 0.021
north-eastern ⇨ NEPL_I	0.054	0.000* - 0.109
south-eastern ⇨ NEPL_I	0.027	0.000* - 0.057
Carpathian ⇨ NEPL_I	0.008	0.000* - 0.022
NEPL_I - nonmigrants	0.722	0.668 - 0.777
recolonized ⇨ north-eastern	0.016	0.000* - 0.036
CentrPL ⇨ north-eastern	0.013	0.000* - 0.034
NEPL_I ⇨ north-eastern	0.018	0.000* - 0.045
south-eastern ⇨ north-eastern	0.016	0.000* - 0.035
Carpathian ⇨ north-eastern	0.010	0.000* - 0.027
north-eastern - nonmigrants	0.927	0.886 - 0.967
recolonized ⇨ south-eastern	0.034	0.000* - 0.077
CentrPL ⇨ south-eastern	0.012	0.000* - 0.034
NEPL_I ⇨ south-eastern	0.020	0.000* - 0.056
north-eastern ⇨ south-eastern	0.043	0.000* - 0.095
Carpathian ⇨ south-eastern	0.014	0.000* - 0.040
south-eastern - nonmigrants	0.877	0.811 - 0.943
recolonized ⇨ Carpathian	0.005	0.000* - 0.015
CentrPL ⇨ Carpathian	0.005	0.000* - 0.014
NEPL_I ⇨ Carpathian	0.037	0.011 - 0.062
north-eastern ⇨ Carpathian	0.008	0.000* - 0.023
south-eastern ⇨ Carpathian	0.006	0.000* - 0.016
Carpathian - nonmigrants	0.939	0.908 - 0.970

Table S4: Migration rates between 11 predefined regions estimated with BayesAss 3.0. 0.000* indicates negative value of lower 95% confidence interval.

	migration rate	95% CI		migration rate	95% CI
WPL_II ➪ WPL_I	0.006	0.000* - 0.016	WPL_I ➪ WPL_II	0.189	0.144 - 0.234
NWPL ➪ WPL_I	0.015	0.000* - 0.033	NWPL ➪ WPL_II	0.050	0.019 - 0.081
VRV ➪ WPL_I	0.003	0.000* - 0.009	VRV ➪ WPL_II	0.006	0.000* - 0.017
CentrPL ➪ WPL_I	0.004	0.000* - 0.011	CentrPL ➪ WPL_II	0.006	0.000* - 0.018
NEPL_I ➪ WPL_I	0.020	0.001 - 0.039	NEPL_I ➪ WPL_II	0.042	0.011 - 0.073
NEPL_II ➪ WPL_I	0.009	0.000* - 0.023	NEPL_II ➪ WPL_II	0.008	0.000* - 0.022
LT ➪ WPL_I	0.004	0.000* - 0.012	LT ➪ WPL_II	0.007	0.000* - 0.020
BY-UA ➪ WPL_I	0.004	0.000* - 0.010	BY-UA ➪ WPL_II	0.006	0.000* - 0.018
SEPL ➪ WPL_I	0.004	0.000* - 0.011	SEPL ➪ WPL_II	0.007	0.000* - 0.021
CarpMTS ➪ WPL_I	0.005	0.000* - 0.014	CarpMTS ➪ WPL_II	0.006	0.000* - 0.018
WPL_I - nonmigrants	0.926	0.895 - 0.958	WPL_II - nonmigrants	0.673	0.661 - 0.685
WPL_I ➪ NWPL	0.209	0.160 - 0.257	WPL_I ➪ VRV	0.183	0.118 - 0.247
WPL_II ➪ NWPL	0.007	0.000* - 0.019	WPL_II ➪ VRV	0.012	0.000* - 0.033
VRV ➪ NWPL	0.006	0.000* - 0.016	NWPL ➪ VRV	0.035	0.000* - 0.072
CentrPL ➪ NWPL	0.007	0.000* - 0.021	CentrPL ➪ VRV	0.011	0.000* - 0.033
NEPL_I ➪ NWPL	0.031	0.000* - 0.067	NEPL_I ➪ VRV	0.023	0.000* - 0.055
NEPL_II ➪ NWPL	0.014	0.000* - 0.035	NEPL_II ➪ VRV	0.012	0.000* - 0.035
LT ➪ NWPL	0.009	0.000* - 0.025	LT ➪ VRV	0.012	0.000* - 0.033
BY-UA ➪ NWPL	0.006	0.000* - 0.017	BY-UA ➪ VRV	0.011	0.000* - 0.032
SEPL ➪ NWPL	0.019	0.000* - 0.040	SEPL ➪ VRV	0.012	0.000* - 0.034
CarpMTS ➪ NWPL	0.007	0.000* - 0.020	CarpMTS ➪ VRV	0.011	0.000* - 0.033
NWPL - nonmigrants	0.687	0.659 - 0.715	VRV - nonmigrants	0.678	0.657 - 0.700
WPL_I ➪ CentrPL	0.152	0.084 - 0.220	WPL_I ➪ NEPL_I	0.145	0.094 - 0.196
WPL_II ➪ CentrPL	0.011	0.000* - 0.032	WPL_II ➪ NEPL_I	0.007	0.000* - 0.022
NWPL ➪ CentrPL	0.017	0.000* - 0.046	NWPL ➪ NEPL_I	0.067	0.002 - 0.132
VRV ➪ CentrPL	0.011	0.000* - 0.032	VRV ➪ NEPL_I	0.006	0.000* - 0.018
NEPL_I ➪ CentrPL	0.021	0.000* - 0.060	CentrPL ➪ NEPL_I	0.007	0.000* - 0.019
NEPL_II ➪ CentrPL	0.016	0.000* - 0.044	NEPL_II ➪ NEPL_I	0.028	0.000* - 0.080
LT ➪ CentrPL	0.023	0.000* - 0.056	LT ➪ NEPL_I	0.009	0.000* - 0.025
BY-UA ➪ CentrPL	0.010	0.000* - 0.031	BY-UA ➪ NEPL_I	0.006	0.000* - 0.019
SEPL ➪ CentrPL	0.039	0.000* - 0.083	SEPL ➪ NEPL_I	0.021	0.000* - 0.046
CarpMTS ➪ CentrPL	0.020	0.000* - 0.052	CarpMTS ➪ NEPL_I	0.007	0.000* - 0.019
CentrPL - nonmigrants	0.678	0.657 - 0.699	NEPL_I - nonmigrants	0.697	0.662 - 0.732
WPL_I ➪ NEPL_II	0.064	0.000* - 0.136	WPL_I ➪ LT	0.006	0.000* - 0.018
WPL_II ➪ NEPL_II	0.008	0.000* - 0.025	WPL_II ➪ LT	0.007	0.000* - 0.021
NWPL ➪ NEPL_II	0.032	0.000* - 0.072	NWPL ➪ LT	0.008	0.000* - 0.024
VRV ➪ NEPL_II	0.007	0.000* - 0.019	VRV ➪ LT	0.005	0.000* - 0.015
CentrPL ➪ NEPL_II	0.007	0.000* - 0.021	CentrPL ➪ LT	0.006	0.000* - 0.017

NEPL_I ➪ NEPL_II	0.027	0.000* - 0.064	NEPL_I ➪ LT	0.019	0.000* - 0.045
LT ➪ NEPL_II	0.029	0.000* - 0.068	NEPL_II ➪ LT	0.172	0.082 - 0.262
BY-UA ➪ NEPL_II	0.007	0.000* - 0.019	BY-UA ➪ LT	0.005	0.000* - 0.016
SEPL ➪ NEPL_II	0.033	0.000* - 0.070	SEPL ➪ LT	0.014	0.000* - 0.036
CarpMTS ➪ NEPL_II	0.009	0.000* - 0.024	CarpMTS ➪ LT	0.008	0.000* - 0.021
NEPL_II - nonmigrants	0.777	0.688 - 0.865	LT - nonmigrants	0.749	0.672 - 0.826
WPL_I ➪ BY-UA	0.019	0.000* - 0.051	WPL_I ➪ SEPL	0.028	0.000* - 0.065
WPL_II ➪ BY-UA	0.017	0.000* - 0.049	WPL_II ➪ SEPL	0.010	0.000* - 0.029
NWPL ➪ BY-UA	0.030	0.000* - 0.084	NWPL ➪ SEPL	0.025	0.000* - 0.061
VRV ➪ BY-UA	0.016	0.000* - 0.047	VRV ➪ SEPL	0.010	0.000* - 0.028
CentrPL ➪ BY-UA	0.018	0.000* - 0.053	CentrPL ➪ SEPL	0.010	0.000* - 0.027
NEPL_I ➪ BY-UA	0.019	0.000* - 0.054	NEPL_I ➪ SEPL	0.013	0.000* - 0.038
NEPL_II ➪ BY-UA	0.109	0.026 - 0.191	NEPL_II ➪ SEPL	0.016	0.000* - 0.046
LT ➪ BY-UA	0.026	0.000* - 0.069	LT ➪ SEPL	0.018	0.000* - 0.048
SEPL ➪ BY-UA	0.025	0.000* - 0.070	BY-UA ➪ SEPL	0.010	0.000* - 0.029
CarpMTS ➪ BY-UA	0.034	0.000* - 0.085	CarpMTS ➪ SEPL	0.012	0.000* - 0.034
BY-UA - nonmigrants	0.687	0.651 - 0.722	SEPL - nonmigrants	0.849	0.784 - 0.913
WPL_I ➪ CarpMTS	0.005	0.000* - 0.015			
WPL_II ➪ CarpMTS	0.004	0.000* - 0.012			
NWPL ➪ CarpMTS	0.009	0.000* - 0.024			
VRV ➪ CarpMTS	0.004	0.000* - 0.012			
CentrPL ➪ CarpMTS	0.005	0.000* - 0.013			
NEPL_I ➪ CarpMTS	0.030	0.005 - 0.055			
NEPL_II ➪ CarpMTS	0.007	0.000* - 0.020			
LT ➪ CarpMTS	0.005	0.000* - 0.015			
BY-UA ➪ CarpMTS	0.004	0.000* - 0.013			
SEPL ➪ CarpMTS	0.005	0.000* - 0.015			
CarpMTS - nonmigrants	0.922	0.889 - 0.955			

Identification of putative wolf-dog hybrids

The whole initial dataset of 894 putative wolf genotypes was screened for the presence of wolf-dog hybrids using software STRUCTURE 2.3.4[1] and DAPC[2], with 50 dog genotypes used as a reference. The dog dataset included both purebred dogs and mixed-breed village dogs, as well as wolf × dog cross-breeds (Saarlos and Czechoslovakian Wolfdog).

In the initial STRUCTURE analysis we identified 33 individuals with ≥0.1 assignment to the dog cluster (Suppl. Fig.S1 A). However, this group included surprisingly high number (21) of Lithuanian individuals that did not reveal any dog-like morphological traits (R. Špinkytė-Bačkaitienė, unpublished data) and from which 20 did not cluster with dogs in DAPC analysis (Suppl. Fig. S1 B). As in the STRUCTURE analysis we observed strong structuring inside the wolf dataset, resulting in a clear separation of the dog cluster only at K≥4, we speculated that results may be biased by unequal sample sizes[3] and presence of large number of closely related individuals[4] in our dataset. Thus, we run an additional analysis including identified putative hybrids, a smaller dataset of wolf genotypes from Mysłajek et al. (manuscript in revision) and the same reference dog genotypes. At K=4, almost all Lithuanian wolves formed a separate cluster and only 13 individuals were still assigned with q ≥0.1 to the dog cluster (Suppl. Fig. S1 C). This included 11 individuals from 3 packs where hybridization was confirmed by field studies (Mysłajek, Nowak, Szewczyk et al., manuscript in preparation) as well as one individual from SEPL and one from Lithuania. All 13 identified crossbred individuals were removed from downstream analyses. Additional DAPC analysis confirmed that the remaining dataset of 881 genotypes did not contain hybrids (Suppl. Fig. S1 D).

Supplementary references

1. Pritchard, J.K., Stephens, M. and Donnelly, P. Inference of population structure using multilocus genotype data. *Genetics* **155**, 945–959. (2000)

2. Jombart, T., Devillard, S. and Balloux, F. Discriminant analysis of principal components: A new method for the analysis of genetically structured populations. *BMC Genetics*. **11**, 94. doi: 10.1186/1471-2156-11-94. (2010)

3. Kalinowski, S. T. The computer program STRUCTURE does not reliably identify the main genetic clusters within species: Simulations and implications for human population structure. *Heredity* **106**, 625–632. doi: 10.1038/hdy.2010.95 (2011)

4. Rodrıguez-Ramilo, S. T., and Wang, J. The effect of close relatives on unsupervised Bayesian clustering algorithms in population genetic structure analysis. *Mol. Eco. Resour*. **12**, 873–884. doi: 10.1111/j.1755-0998.2012.03156.x (2012)
